# LW-PWDNet: a lightweight and cross-terrain adaptive framework for early pine wilt disease detection

**DOI:** 10.3389/fpls.2025.1687742

**Published:** 2025-10-30

**Authors:** Yongkang Hu, Fang Wang

**Affiliations:** College of Electronic Engineering, Nanjing XiaoZhuang University, Nanjing, China

**Keywords:** pine wilt disease, computer vision, lightweight, object detection, UAV

## Abstract

Pine wilt disease (PWD) poses a severe threat to forest ecosystems due to its high infectivity and destructive nature. Early identification of PWD-infected pines is critical to curbing disease spread and safeguarding forest resources. In order to timely detect and prevent the spread of PWD and meet the requirements of edge computing devices for real-time performance and computational efficiency, this paper proposes a lightweight model LW-PWDNet. The backbone network reconstructs HGNetV2 to achieve efficient feature extraction. It decomposes traditional convolutions into more lightweight feature generation and transformation operations, reducing computational cost while retaining discriminative power. The feature fusion layer reconstructs the path aggregation network based on RepBlock and multi-scale attention mechanism, capturing fine-grained details of small lesions, so as to better capture the detailed features of small targets. At the same time, this paper designs a lightweight D-Sample down-sampling module in the feature fusion layer to further improve the model's detection ability for multi-scale targets. Finally, this paper designs a lightweight prediction layer LightShiftHead for this model. By strengthening the local feature expression, the detection accuracy of PWD in small targets is further improved. A large number of experimental results show that LW-PWDNet maintains a high detection accuracy of mAP 89.7%, while achieving low computational complexity of 5.6 GFLOPs and only 1.9M parameters, as well as a high inference speed of 166 FPS when tested on an NVIDIA RTX 4070 GPU with a 13th Gen Intel(R) Core(TM) i7-13700KF processor, using PyTorch 2.0.1 and CUDA 12.6, based on Python 3.9. This model can provide an efficient and lightweight detection solution for PWD in resource-constrained scenarios such as unmanned aerial vehicle inspections.

## 1 Introduction

Pine wilt disease (PWD) is caused by a wood pathogen carried by the pinewood nematode. The disease has a short incubation period and is highly contagious. It only takes 40 days for a pine tree to die after being infected. Can potentially destroy an entire pine forest within 3–5 years under favorable conditions (Li et al., 2022a). PWD has become one of the most destructive forest diseases in the world. The disease was first discovered in North America in the early 20th century and has since spread rapidly to Asia and Europe (Back, 2020). In China, PWD was first discovered in 1982 in the black forest of the Dr. Sun Yat-sen Mausoleum in Nanjing, and it has spread to many regions across the country at an alarming rate, increasing from a few hundred hectares in the 1980s to over 2 million hectares in 2024 (Hao et al., 2022).

PWD is characterized by rapid spread, high mortality, and costly control, making early detection essential. Monitoring the early stages of PWD is therefore crucial. According to a refined economic assessment conducted at the subcompartment scale (Liu et al., 2023), PWD resulted in a total loss of approximately USD 7.4 billion in China in 2020, including USD 1.11 billion in direct economic losses and USD 6.29 billion in ecosystem service losses. Recent work by Liu et al. (Liu et al., 2024) also introduced the Clusterformer segmentation framework, which demonstrated the potential of UAV-based deep models for PWD-related tree identification, highlighting the necessity of integrating lightweight segmentation and detection mechanisms. A considerable portion of the direct costs originated from large-scale quarantine logging, transportation, incineration, and reforestation operations. Studies have shown that timely detection and removal of infected trees during the early infection stage can reduce the number of felled trees by over 40%, substantially alleviating the burden on forestry resources and management budgets.

Ground surveys are labor-intensive and often miss early infections, and frequently fail to detect early infections (Li et al., 2023). In addition, the external manifestations of pine trees infected with PWD, such as needle discoloration and canopy thinning, usually only appear after irreversible damage has occurred (Shi et al., 2024).

In recent years, with the development of remote sensing technology, it has the advantages of wide coverage, high temporal resolution, short revisit period, and wide spatial coverage, providing a solid technical foundation for the timely monitoring of PWD. Currently, the monitoring of forest pests and diseases using remote sensing technology mainly falls into two directions: one is through satellite remote sensing monitoring, and the other is through unmanned aerial vehicle (UAV) remote sensing monitoring (Li et al., 2022b).

However, satellite remote sensing has many limitations in the monitoring of PWD. Its spatial resolution is relatively low (usually 10–30 meters), making it difficult to accurately identify individual trees in the early stage of infection, especially in the stage when the symptoms are not yet obvious (Cai et al., 2023; Xie et al., 2024). In addition, satellite images are greatly affected by the fixed revisit cycle and weather factors, and it is difficult to meet the needs of continuous and high-frequency monitoring. Most satellites use vertical downward-looking imaging, lacking side-looking angle information, making it difficult to extract key features such as needle discoloration or branch structure (Ye et al., 2025). At the same time, the scale of satellite images is relatively coarse, making it difficult to label diseased trees and conduct ground verification, which is not conducive to constructing a high-quality deep learning training dataset. In contrast, UAV remote sensing has higher spatial resolution and greater deployment flexibility, and can more effectively achieve early and fine-grained detection of PWD (Sun et al., 2022; Ye et al., 2023; Du et al., 2024; Yao et al., 2024). In particular, Yao et al. (Yao et al., 2024) proposed the Pine-YOLO detection framework, which improved UAV-based monitoring performance through task-specific architectural refinement, laying a foundation for subsequent lightweight detection research.

At present, some studies on using UAV remote sensing monitoring adopt traditional machine learning or feature classification strategies. For example, Thapa et al (Thapa et al., 2024). constructed a multi-stage classification structure based on deep metric learning, and improved the recognition robustness through feature embedding, which has certain prior dependence. A large number of studies use deep learning models to identify diseased plants. For example, the joint deep object detection model proposed by Wu et al (Wu et al., 2024), the improvement of the YOLO model for the infection stage by Wu et al (Wu et al., 2023), the use of the YOLOv4 structure by Zhang et al (Zhang et al., 2024), and the detection model combining GoogLeNet by Zhu et al (Zhu et al., 2023). In addition, multiple studies have begun introducing attention mechanisms, ViT modules, and hybrid CNN–Transformer structures for vegetation health monitoring. For instance, Wang et al. (Wang et al., 2024a) proposed FireViTNet integrating ViT and CNNs for forest fire segmentation, while Chen et al. (Chen et al., 2024) developed TeaViTNet using multiscale attention fusion for pest detection in tea leaves, both illustrating the transferability of such architectures to forest pathology contexts. Similarly, Wang et al. (Wang et al., 2024b) and Jin et al. (Jin et al., 2024) explored lightweight vision frameworks (SWVR and Fire-in-Focus) for efficient forest fire recognition, further validating the practical value of lightweight visual perception in UAV environmental monitoring. These methods perform well in terms of accuracy, but the network structure is complex, the inference efficiency is low, and it is difficult to meet the requirements of edge deployment. Recent work such as Li & Peng (Li & Peng., 2024) and Lin, Xiao, & Lin (Lin, et al., 2025) also focused on lightweight YOLOv8 optimization in industrial and agricultural detection, respectively, suggesting a general trend toward efficient inference networks. Similarly, the multi-scale channel adaptive network designed by Ren et al (Ren et al., 2022), the local feature extraction method based on Mask R-CNN by Wu and Jiang (Wu and Jiang, 2023), the improved Mask R-CNN by Hu et al (Hu et al., 2022), and the performance evaluation of deep networks such as ResNet and DenseNet by Zhi et al (Zhi et al., 2024) all belong to non-lightweight structures, which are suitable for scenarios where accuracy is prioritized but not friendly to resource-constrained environments.

At the same time, in the face of the growing demand for lightweight models in drone monitoring, in order to achieve both low computational overhead and high recognition accuracy, some research focuses on the integration of network structure lightweighting and attention mechanisms. For example, in response to the problems of high forest density and small target scale, Yu et al (Yu et al., 2023). proposed a shallow weighted feature enhancement network (SPWFEN), which effectively improved the recall rate of small target detection. Gu et al (Gu et al., 2025). developed DEMNet, a lightweight detection framework designed for tea leaf blight in slightly blurry UAV remote sensing images, demonstrating the importance of specialized designs for small and visually indistinct targets. Similarly, Liu et al (Liu et al., 2024). proposed Clusterformer, a Transformer-based segmentation framework tailored for pine tree disease identification in UAV remote sensing images, which enhanced fine-grained feature representation and showed strong adaptability to complex forest backgrounds. Dong et al (Dong et al., 2024). introduced an attention module and a multi-task loss function into YOLOv5, Han et al (Han et al., 2022). proposed a network combining a Gaussian kernel and a multi-scale spatial attention mechanism, Bai et al (Bai et al., 2025). constructed a detection structure suitable for complex scenes based on the lightweight Mamba model and the attention module, Xu et al (Xu et al., 2023). fused color features and spatial attention for the identification of discolored diseased trees, and Zhang et al (Zhang et al., 2023). optimized the YOLOv5 backbone network through the attention mechanism, all achieving a good balance between model performance and computational efficiency. Chen et al (Chen et al., 2024). Chen et al. (2025) innovatively integrated the visual Transformer (ViT) and CNN to construct PWDViTNet, taking into account the detection of weak-texture diseased plants at long distances and the lightweight deployment of the network, showing good prospects.

Furthermore, recent studies have extended PWD detection to multi-stage joint frameworks and fine-grained feature fusion. For instance, Zhou et al. (Zhou et al., 2025) proposed a PWD-lightweight and feature fusion network for multi-stage detection, while Wang et al. (Wang et al., 2025) introduced a hierarchical attention and feature enhancement network for multi-scale small targets, both of which emphasize efficient spatial-semantic aggregation.

From a methodological perspective, the growing body of work by Lin and collaborators (Lin & Tang, 2021a; Lin & Tang, 2021b; Lin et al., 2022; Lin, Qian & Di, 2023a; Lin et al., 2023b) on intelligent optimization, cloud-based detection, and lightweight design provides a theoretical foundation for integrating multi-objective optimization and feature extraction into UAV-based lightweight frameworks such as LW-PWDNet.

In summary, although non-lightweight models have advantages in detection accuracy, their deployment flexibility and resource adaptability are poor. In contrast, the small target detection method based on lightweight structures and attention mechanisms provides a more feasible solution for the efficient, low-cost, and edge monitoring of PWD, and it is also the key research direction for researchers at present.

Considering that in practical applications, the monitoring of PWD relies on real-time detection and deployment by edge devices such as drones. Therefore, the lightweighting of the model is crucial for reducing computational costs, improving inference speed, and adapting to resource-constrained devices. To meet this requirement, this study proposes LW-PWDNet, a collaborative lightweight architecture built upon the PWD-EFC, D-Sample, and LightShiftHead modules. This design not only differs from conventional backbone-pruning strategies seen in lightweight YOLO variants but also incorporates attention-guided fusion and parameter-efficient optimization inspired by Lin et al. (Lin et al., 2025) and related works.

First, this paper proposes a lightweight detection model, LW-PWDNet. Its core innovation lies in the collaborative lightweight design of the PWD-EFC feature fusion module, the D-Sample multi-scale down-sampling module, and the LightShiftHead prediction layer. The organic combination of these three not only effectively reduces the model's parameter quantity (Parameters reduced to 1.9 M) and computational amount (GFLOPs reduced to 5.6), but also enhances the feature extraction and localization capabilities for small PWD targets in the early stage. This is significantly different from the single optimization idea of existing lightweight YOLO frameworks that only rely on backbone cropping or operator replacement.

Secondly, a dataset containing 8,000 PWD pine tree images in different scenarios was constructed, and comparative experiments were carried out on this dataset with various lightweight baseline models such as YOLOv5n and YOLOv10n. The results show that LW-PWDNet has a 2.8% improvement in the mAP indicator compared to mainstream lightweight detection frameworks, verifying the performance advantage of the proposed collaborative lightweight design in the PWD small target detection task.

Finally, this paper further verifies the adaptability of LW-PWDNet on UAV edge devices, and completes practical deployment and detection experiments. It shows that this model not only has academic method innovation, but can also provide a practical technical support for large-scale and real-time monitoring of forestry PWD.

## 2 Materials and methods

### 2.1 Study area

**Figure 1:**
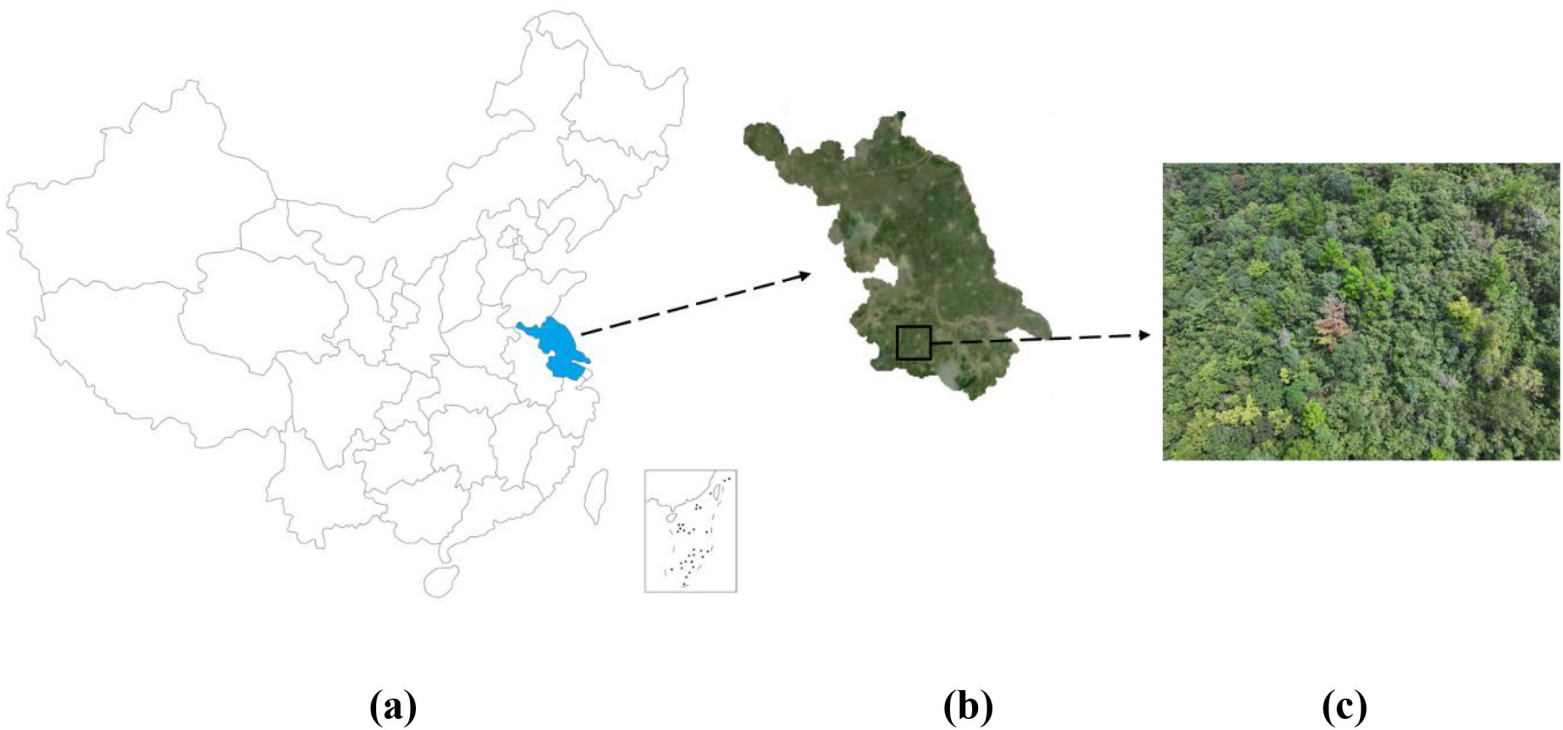
**(a)** shows the location of Jiangsu Province in China, **(b)** shows the location of Liyang City in Jiangsu Province and satellite images of Liyang City, and **(c)** shows images of field surveys by UAVs.

Jiangsu Province (latitude 30°45′-35°20′N, longitude 116°18′-121°57′E) is located in the eastern coastal area of China, at the lower reaches of the Yangtze River and on the shore of the Yellow Sea (**Figure 1**). Jiangsu has a gently sloping terrain from southwest to northeast. The landform is mainly plain, with hills and low mountains. The Taihu Lake Basin in the south and the hilly areas in the west are relatively rich in forestry resources. The forest coverage rate of the whole province is about 24.03%, and the area of coniferous forest accounts for 26.17% of the total forest area of the province. The forest distribution in Jiangsu is mainly concentrated in the hilly areas of southern Jiangsu, the Ningzhen Mountains and the areas along the rivers and lakes.

The study area is Liyang City (latitude 31°21′-31°43′ N, longitude 119°05′-119°29′ E), located in the southern part of Jiangsu Province and the southwestern part of Changzhou City, at the northern foot of the Tianmu Mountains in the hilly area. The terrain of Liyang City generally slopes from the southwest to the northeast. It belongs to the transitional landform of low mountains, hills and plains, and has typical southern forest ecological characteristics. The vegetation types are diverse, covering coniferous forests, evergreen broad-leaved forests, deciduous broad-leaved forests and artificial mixed forests, with a complex forest structure. The forest land area of the whole city is about 2,200 square kilometers, and the forest coverage rate exceeds 40%. Among them, coniferous forests are widely distributed in mountainous and gentle slope areas. Liyang is one of the key epidemic areas of PWD in Jiangsu Province. In recent years, the infection pressure has persisted. It is representative, typical and has the value of continuous monitoring. It is an ideal area for carrying out long-term monitoring and model optimization research of PWD.

### 2.2 Data collection

We acquired images with a DJI Mavic 3 UAV and the UAV configuration is shown in **Table 1**.

**Table 1:** UAV configuration.

Parameter name	Parameter value
Name of UAV	DJI Mavic 3
Aerial Camera	Hasselblad L2D-20c
Sensor Size	17.3mm×13.0mm (4/3 CMOS sensor)
Lens focal length	24mm (equivalent)

During the image acquisition process, manually selected the flight range and take pictures at equal-distance intervals to ensure the uniformity and comprehensiveness of data collection. In order to adapt to the complex terrain undulations and diverse vegetation densities in forest areas, this study adopted a planar route planning to effectively cover the entire acquisition area while minimizing flight path overlap.

**Figure 2:**
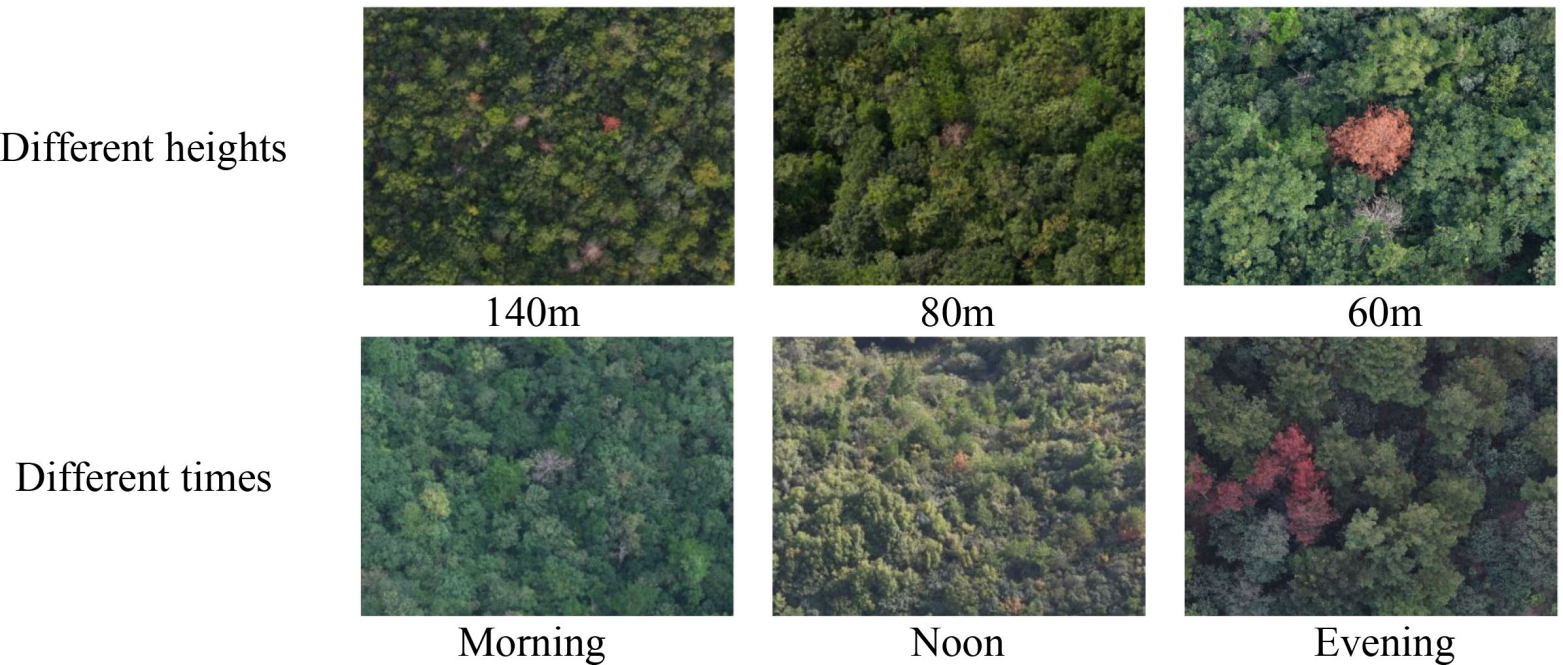
UAV images acquisition.

To enrich the diversity and comprehensiveness of the PWD dataset, this chapter collected PWD images at different time periods (morning, noon, and dusk), different shooting heights (140 meters, 80 meters, and 60 meters above the ground), and different infection levels, ensuring that the dataset contains PWD information under various environmental conditions. After more than 30 flight shootings, a total of 11,126 PWD images were obtained in this chapter. Some example image data are shown in **Figure 2**:

This paper uses a DJI drone for image acquisition. The resolution of the acquired images is 4032×3024. High-resolution images will occupy a large amount of storage space and computing resources during training, increasing processing time and hardware burden. In addition, although high-resolution images can provide rich detail information, they may also contain redundant and invalid information irrelevant to the detection task of PWD-infected trees. For example, the images may include a large amount of background, irrelevant trees, distant objects, or detailed textures. These contents not only increase the computational burden but may also cause noise interference to the target detection algorithm, affecting the detection accuracy of the algorithm.

**Figure 3:**
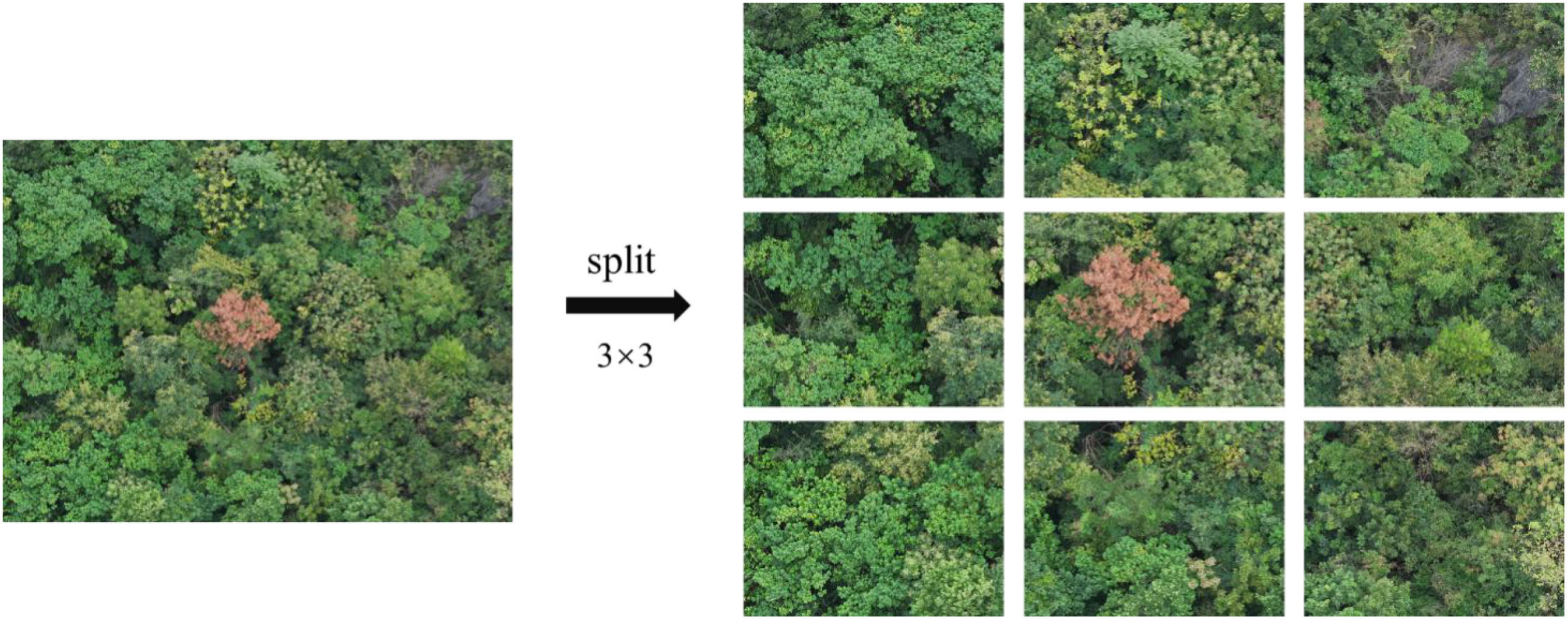
Image segmentation.

Therefore, in order to improve the efficiency of data processing and reduce the consumption of computing resources, this paper evenly divides the original images at a ratio of 3×3. The image resolution is reduced to 1333×1000, which not only retains the necessary detail information but also significantly reduces the amount of data, thus reducing the computational burden of model training. **Figure 3** shows the segmented sample images.

### 2.3 Dataset production

**Figure 4:**
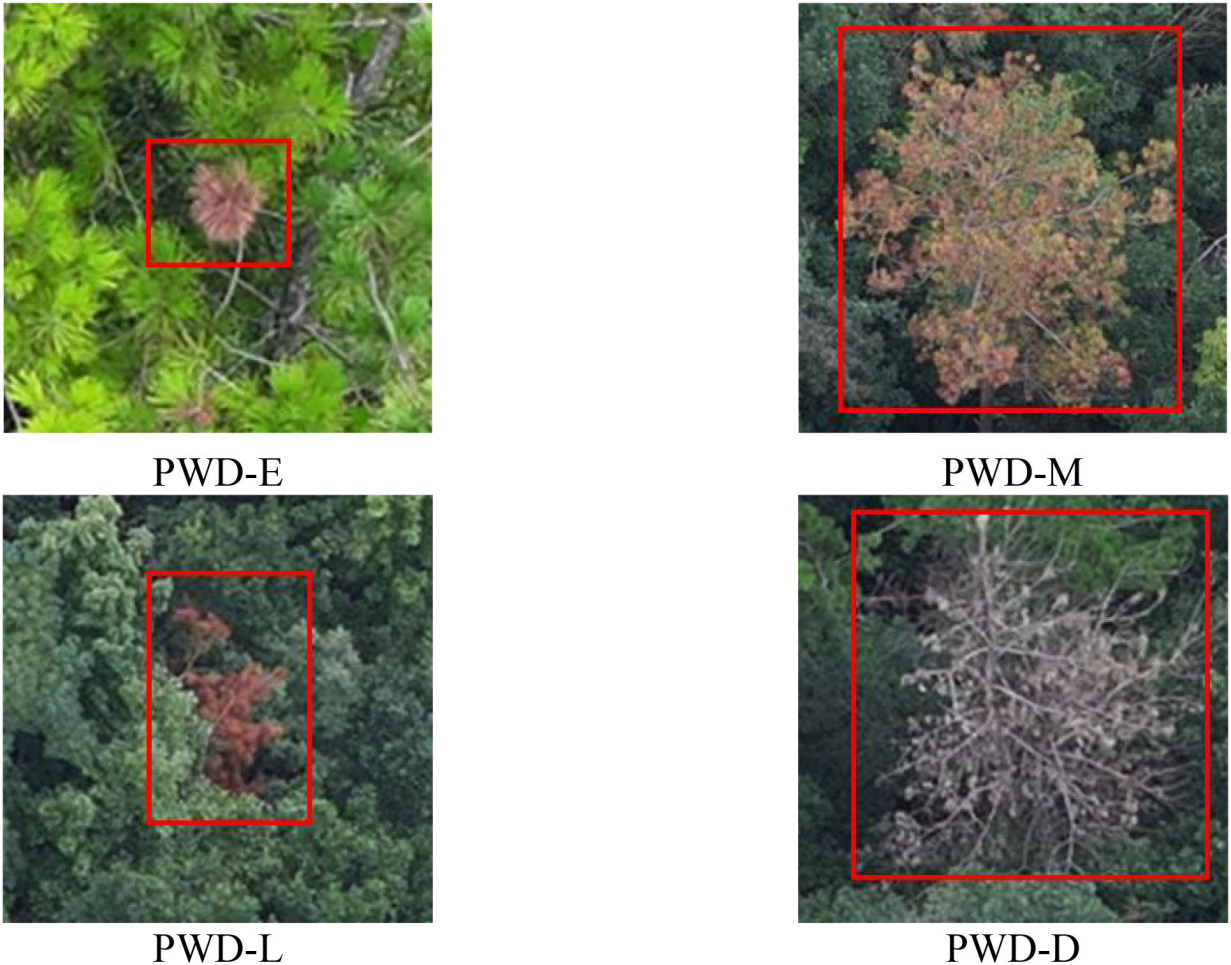
The 4 stages of PWD.

Based on the clinical manifestations and image visual characteristics of PWD, this paper divides the infection stages of PWD into four stages: early stage, middle stage, late stage, and dead tree, as shown in **Figure 4**. In the early infection stage, it is usually manifested as sporadic yellowing of needles, which generally starts from the top of the crown or a certain part and gradually spreads around. In the middle infection stage, the needles of the pine tree begin to wither on a large scale, and the infected parts usually turn yellowish-brown or grayish-yellow. In the late infection stage, the needles of the entire pine tree show obvious yellowing and withering, the vitality of the pine tree gradually declines, and the trunk completely loses its green color, usually showing dark brown. In the dead tree stage, the pine tree almost completely loses its needles and branches, showing obvious rot or death, and the color of the trunk changes to gray or dark brown.

To improve the quality of the dataset, this study screened the cut images, removed redundant images, and finally retained 8000 valid data, and annotated them. After screening redundant images and validating annotation quality, we finally retained 8,000 valid samples, with the number of labeled samples for each PWD infection stage (Early, Middle, Late, Dead) detailed in **Table 2**. The dataset was split into training, validation, and test sets at an 8:1:1 ratio, ensuring the independence of each subset for reliable model evaluation.

**Table 2:** The number of labels samples in the dataset.

Dataset Split	PWD-E	PWD-M	PWD-L	PWD-D
Train	7215	1892	3798	2681
Test	2397	615	1289	857
Val	2438	629	1312	876
Total	12050	3136	6399	4413

The dataset uses the LabelImg tool to accurately annotate the targets in the RGB images captured by the drone. First, use a rectangular box to frame the specific location of the diseased area. Each rectangular box annotation represents a target in the image. The four vertices of the annotation box are clearly specified by coordinate values, thus accurately describing the position and size of the target in the image. Then, assign a corresponding category label to each rectangular box annotation. Pine trees with early-stage lesions are labeled as PWD-E (Early), those with mid-stage lesions are labeled as PWD-M (Middle), those with late-stage lesions are labeled as PWD-L (Late), and dead pine trees are labeled as PWD-D (Dead).

### 2.4 LW-PWDNet model construction

**Figure 5:**
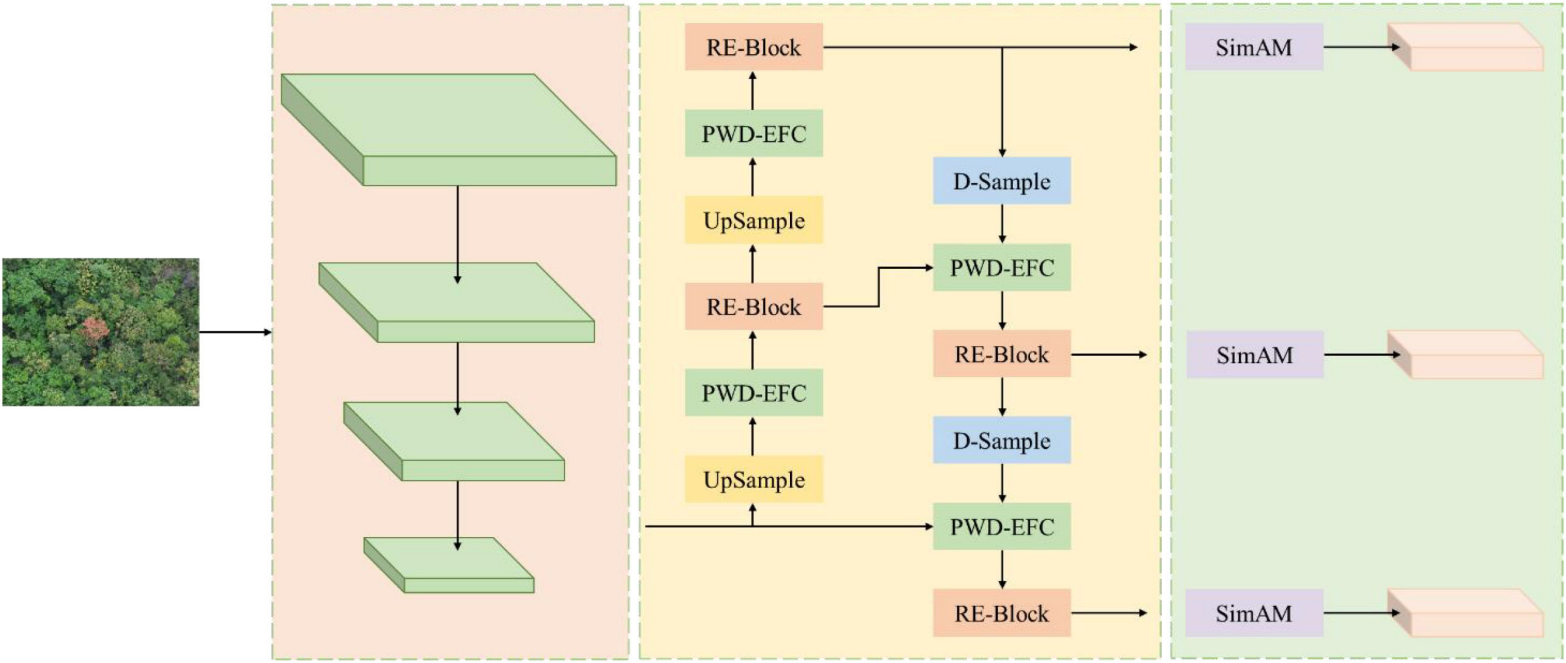
LW-PWDNet model structure.

The structure of the lightweight PWD detection network LW-PWDNet proposed in this paper is shown in **Figure 5**. The model mainly consists of a backbone network layer, a feature fusion layer, and a prediction layer. In order to meet the resource limitations of edge devices and achieve efficient and accurate detection of small-target PWDs, a lightweight backbone network GH-Backbone was designed, and the HGNetV2 network structure was reconstructed. While reducing the computational complexity, the perception ability of the PWD disease area was improved. For the feature fusion layer, based on enhancing the inter-layer feature correlation, a lightweight feature fusion module PWD Enhanced Inter-Layer Feature Correlation (PWD-EFC) was designed to improve the feature expression ability and detection accuracy. In addition, a D-Sample downsampling module with a dual-branch structure was designed to reduce information loss and further improve the detection accuracy of small-target PWDs. Finally, at the prediction layer, an ultra-lightweight detection head LightShiftHead was constructed based on depthwise separable convolution (DWConv) and sparse convolution (SPConv) to reduce redundant calculations, further optimize the overall efficiency of the model, and ensure the accurate detection of small-target PWDs.

### 2.5 Backbone network

Lightweight backbone network design should, on the basis of ensuring accuracy, effectively improve the model's inference efficiency, reduce resource consumption, and thus better meet the real-time detection requirements on edge devices. In order to meet these requirements, this paper draws on the structure of the backbone network HGNetV2 of RT-DETR deep learning model (Zhao et al., 2024), constructs GHBackbone as the backbone network of LW-PWDNet model, and optimizes it with GhostConv (Han et al., 2020).

**Figure 6:**
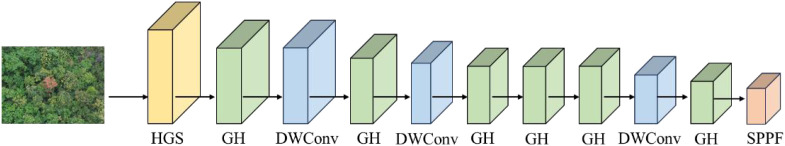
GH-backbone framework.

GH-Backbone consists of High-Resolution group Stem(HGS), GhostHGB(GH), DWConv (Zhang et al., 2023) and SPPF (Li et al, 2024) modules, and its architecture is shown in **Figure 6**.

**Figure 7:**
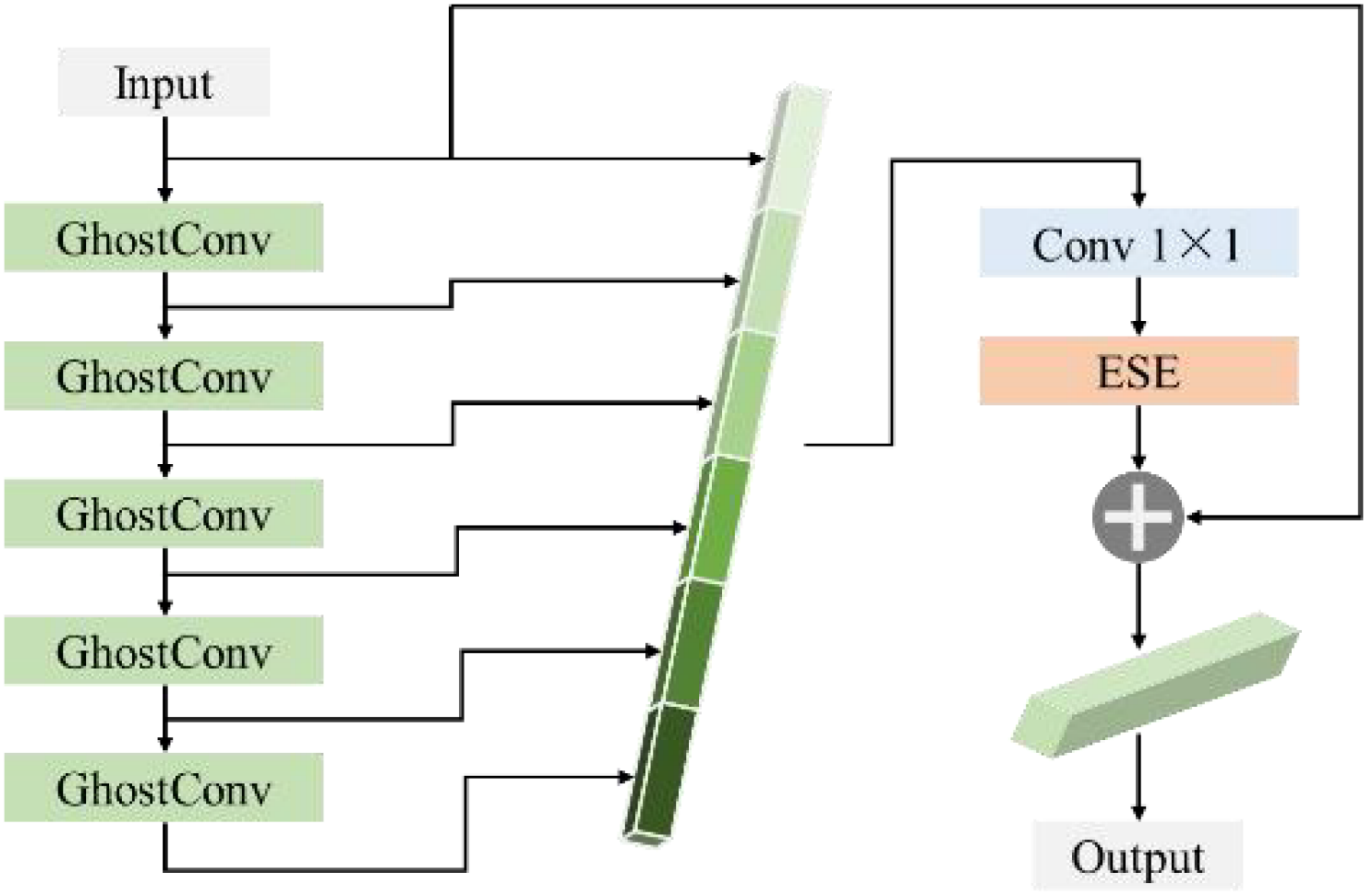
GH modular structural framework.

In the GH-Backbone, the size of the input image is 640×640. The HGS module first preprocesses the images of PWD-infected trees captured by drones at high altitude, The HGS module preprocesses the images, converting them into low-level feature maps for the subsequent network to perform deeper feature extraction. On this basis, in the GH-Backbone structure, this paper adopts an alternating stacking of GH and depthwise separable convolutions to improve the model's feature extraction ability and computational efficiency. The features processed by HGS first undergo preliminary feature enhancement through GH, and then enter the DWConv to reduce the computational complexity and further improve the feature representation ability. The alternating action of GH and DWConv enables the network to efficiently extract and fuse information at different scales, gradually enhancing the detection ability for small targets of PWD. The GH structure uses residual connections to connect multiple GhostConvs to enhance the fluidity of PWD features, improve gradient transmission, alleviate the problem of gradient vanishing in the training of deep networks, and at the same time improve the model's detection ability for PWD disease targets of different scales. Its structure is shown in **Figure 7**.

The Spatial Pyramid Pooling-Fast (SPPF) module at the end of the backbone network expands the receptive field with low computational cost by introducing multiple pooling operations at different scales, reducing the computational burden while maintaining detection accuracy. Compared with the traditional Spatial Pyramid Pooling (SPP) module, SPPF uses fewer convolutional calculations, improving the inference efficiency of the model on edge devices. In addition, SPPF can effectively integrate deep-feature information, enhancing the model's perception ability of the target area of PWD, thus improving the robustness and accuracy of small-target detection.

### 2.6 Feature fusion layer

In the LW-PWDNet, the feature fusion layer adopts a PAN-based structure to fully integrate the features of PWD targets at multiple scales, improving the model's perception ability for disease regions of different scales. In traditional feature pyramids, such as FPN, the fusion of multi-scale features mainly uses a top-down approach, in which deep-layer semantic features gradually enhance the spatial detail information of shallow layers (Li et al., 2024). PAN further introduces a bottom-up path enhancement mechanism, enabling shallow-layer features to also influence deep-layer features in the reverse direction, thus improving the detection model's representation capability for targets of different scales, especially the recognition of small targets. Compared with other alternatives such as BiFPN, PAN was selected for its superior balance between detection accuracy and computational efficiency in small-target detection tasks. In addition, considering the challenges of small-target feature loss in PWD detection, insufficient feature representation of multi-scale disease regions, limited computing resources, and high inference speed requirements, this study further optimizes computational efficiency while ensuring that the feature fusion layer maintains effective information interaction, making it suitable for edge computing devices.

**Figure 8:**
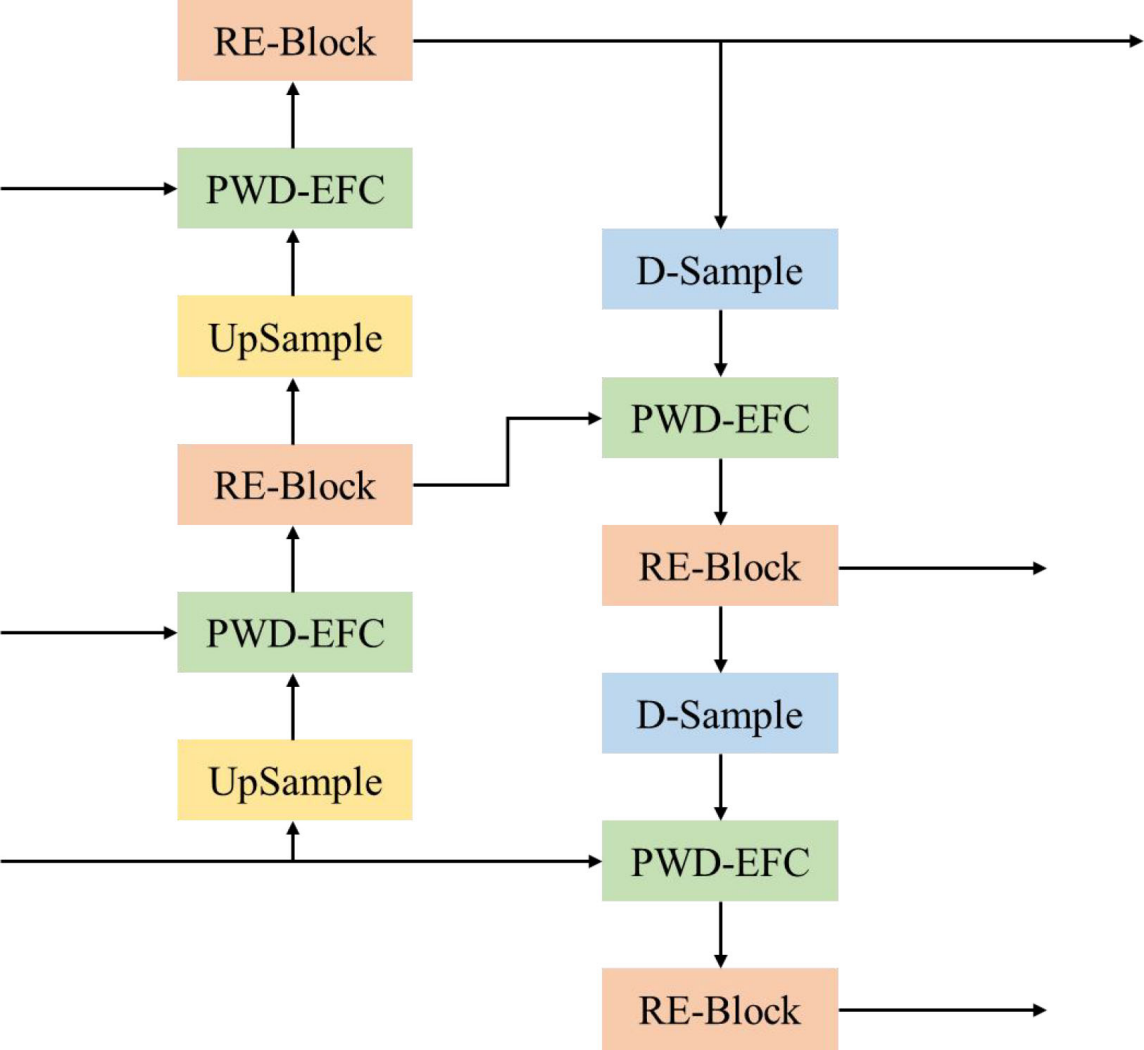
Structural framework of feature fusion layer.

The architecture of the feature fusion layer of the LW-PWDNet model is shown in **Figure 8**. Specifically, in this paper, a RE-Block is constructed based on the RepBlock and the multi-scale attention mechanism Efficient Multi-scale Attention (EMA) (Ouyang et al., 2023) to improve the efficiency of multi-scale information interaction. Among them, the RepBlock adopts a re-parameterizable structure. In the inference stage, the convolutional branches can be merged, reducing the computational complexity and improving the inference speed of the model, which is suitable for edge computing deployment. The EMA mechanism can adaptively adjust the feature weights at different scales, enabling the model to pay more attention to the fine-grained features of the PWD area while effectively suppressing the interference of background noise. In addition, a lightweight feature fusion module, the PWD-EFC module, is designed in the feature fusion layer to enhance the feature correlation between different layers. In order to further optimize the computational cost and effectively improve the feature representation ability, this paper designs and constructs a D-Sample down-sampling module to optimize the information transfer between feature layers.

### 2.7 Module

#### 2.7.1 RE-block module

**Figure 9:**
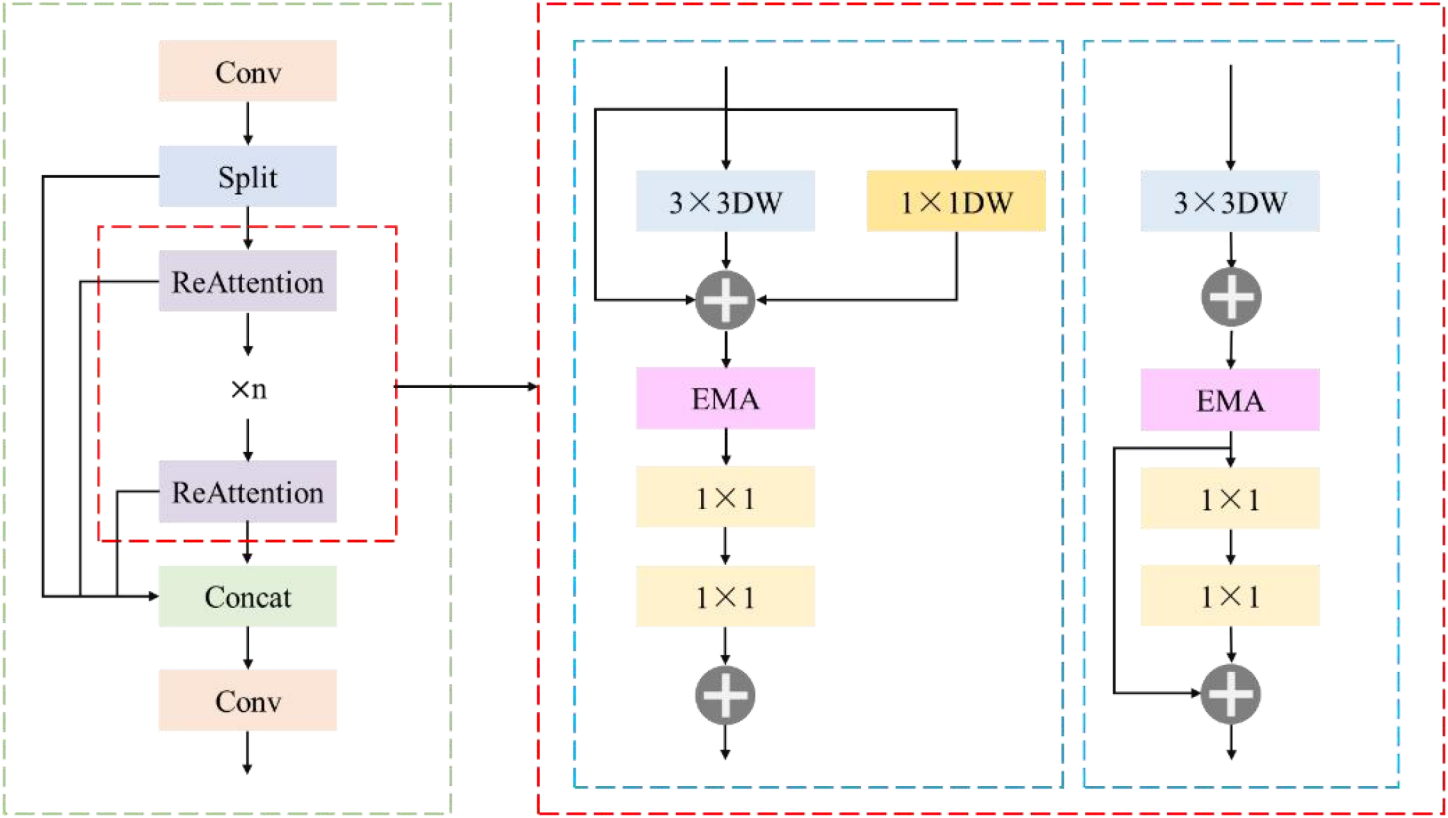
RE-block structural framework.

The core design of RE-Block is the ReAttention module, which consists of RepBlock and EMA. Its structure is shown in **Figure 9**. During the training phase, RepBlock adopts a dual-branch DWConv structure to improve the feature extraction ability and enhance the model's expressive power. During the inference phase, in order to reduce the computational cost and improve the inference efficiency, the method of structural re-parameterization is adopted to merge the two parallel DWConv branches in the training phase into an equivalent single DWConv. Specifically, this merging operation is based on the method of convolutional weight fusion, that is, the weights calculated by the two DWConvs are weighted and merged, so that only one DWConv operation needs to be performed during inference, without the need for additional multi-branch calculations. This not only reduces the amount of calculation but also retains the effective features learned by the multi-branch structure during the training phase, ensuring that LW-PWDNet still has a strong feature extraction ability. DWConv decouples the convolution into depthwise and pointwise operations. Channel-wise convolution independently performs spatial convolution operations on each input channel, retains fine-grained local spatial information, and reduces computational redundancy. Subsequently, point-wise convolution performs information interaction between channels on the output of channel-wise convolution, enhances the expressive power of features, and ensures that the target information of PWD at different scales can be effectively integrated.

Although the RepBlock effectively reduces the computational cost and streamlines the parameter count of the LW-PWDNet model, relying solely on this module will inevitably lead to a loss in PWD detection accuracy. Especially in the task of detecting small targets of PWD, fine-grained feature representation is crucial for high-precision target recognition. To compensate for the loss in detection accuracy, this paper introduces the EMA attention mechanism. The EMA module is ingeniously designed to utilize a grouping structure that does not require dimensionality reduction, combined with cross-space learning techniques, effectively capturing both short-term and long-term dependencies in the image. By grouping the channel dimension into multiple sub-features, the EMA module can enhance the uniform distribution of spatial semantic information in the feature map while ensuring the retention of information in each channel. This design enables effective aggregation of features at different scales, improving the detection accuracy of small PWD targets.

Suppose the arbitrary input feature map is:


(1) $$\begin{array}{c} \text{X}\in {\mathbb{R}}^{\text{C}\times \text{H}\times \text{W}} \end{array}$$

Among them, C, H, and W represent the number of channels, height, and width of the input feature map, respectively. EMA learns different semantic information by dividing the feature map into G sub-features in the cross-channel dimension. The group style represented by each sub-feature can be defined as:


(2) $$\begin{array}{c} X=\left[{\text{X}}_{0},{\text{X}}_{1}\ldots \ldots {\text{X}}_{\text{G}-1}\right] \end{array}$$

In order to effectively capture the interdependencies between channels and reduce computational overhead, EMA performs global average pooling operations along H and W respectively in the 1×1 branch, thereby encoding the channel features. Specifically, two one-dimensional global average pooling operations are employed to enhance the inter-channel correlation. In the 3×3 branch, multi-scale features are captured by stacking a single 3×3 convolutional kernel. Subsequently, before the channel feature joint activation mechanism, the one-dimensional feature encoding vector obtained through global average pooling is reshaped and adjusted in shape to ensure its suitability for subsequent processing. This process can be represented by the formula:


(3) $$\begin{array}{c} {\mathbb{R}}_{3}^{1\times \text{C}//\text{G}}\times {\mathbb{R}}_{1}^{\text{C}\times \text{C}//\text{G}} \end{array}$$

On this basis, a second spatial attention map that retains precise spatial location information was further derived. Subsequently, the output feature maps within each group were combined with the two generated spatial attention weight values. The Sigmoid function was used to capture the pairwise relationships at the pixel level and strengthen the global context information of all pixels.

#### 2.7.2 PWD-EFC

**Figure 10:**
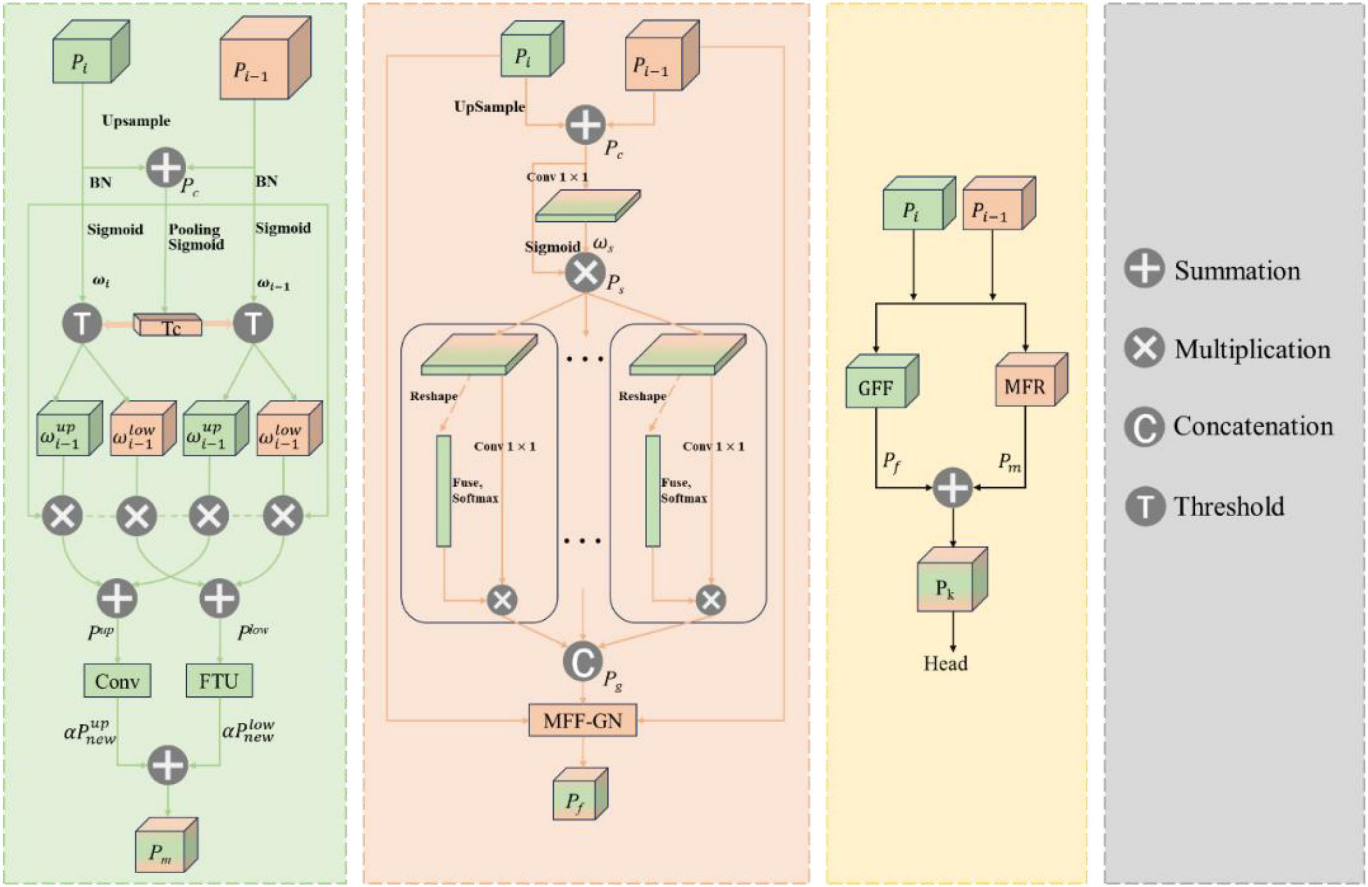
PWD-EFC structural framework.

To improve the feature fusion ability of LW-PWDNet in small object detection, this paper designs a lightweight feature fusion module called PWD-EFC, whose structure is shown in **Figure 10**. PWD-EFC is used to strengthen the context connection and semantic consistency among feature maps at different levels, and effectively improve the detection performance of small objects without significantly increasing the number of parameters. This module can achieve faster inference speed and lower energy consumption while ensuring accuracy, and improve the recognition accuracy and efficiency of early PWD.

The PWD-EFC consists of two main components: the Grouped Feature Focus (GFF) unit and the Multi-level Feature Reconstruction (MFR) module. The GFF module aims to enhance the contextual correlation among feature maps of different scales. Its main idea is to simulate a process similar to the attention mechanism, extracting key information regions through spatial focusing, channel grouping, and spatial normalization operations. The MFR separates strong and weak spatial information and uses lightweight convolutional modules to achieve precise feature transformation. This approach reduces the extraction of irrelevant information while preserving the key details of small objects in deep networks.

The design concept of the PWD-EFC module is mainly reflected in three aspects: feature compression, feature recombination, and feature fusion. The first part is the grouped feature attention mechanism. Its core idea is to improve the correlation between features at different levels by enhancing spatial attention and channel context information. At the input end, the PWD-EFC module receives two feature maps from different layers, denoted as $x_{1}\in {\mathbb{R}}^{C\times H\times W}$ and $x_{2}\in {\mathbb{R}}^{C\times H\times W}$ . In order to unify the dimensions and enhance the representation ability, a 1×1 convolution and batch normalization are first applied to both of them, and an attention weight map is generated through the Sigmoid function:


(4) $$\begin{array}{c} {\text{w}}_{1}=\sigma \text{BN}\left({\text{Conv}}_{1}\left({\text{x}}_{1}\right)\right){\text{, w}}_{2}=\sigma \text{BN}\left({\text{Conv}}_{2}\left({\text{x}}_{2}\right)\right) \end{array}$$

The above operation can be regarded as an explicit spatial attention mechanism for capturing the importance of key region positions. After obtaining the two weighted feature maps, the PWD-EFC module performs channel-level fusion to generate a global feature representation:


(5) $$\begin{array}{c} {\text{X}}_{global}={\text{Conv}}_{1}{\text{(x}}_{1})+{\text{Conv}}_{2}{\text{(x}}_{2}) \end{array}$$

Subsequently, in order to explore more fine-grained channel context relationships, PWD-EFC divides the global feature map into g groups (g denotes the number of groups, empirically set to 4 in this study) along the channel dimension, that is, ${\text{X}}_{global}=\{{\text{X}}^{\left(1\right)},{\text{X}}^{\left(2\right)},\ldots ,{\text{X}}^{\left(g\right)}\}$ . A lightweight convolutional operation is applied to each group of features to obtain the intra-group context interaction features. By normalizing each group of features $X^{\left(i\right)}$ and using Softmax to generate normalized attention weights:


(6) $$\begin{array}{c} {\text{Y}}^{\left(i\right)}={\text{X}}^{\left(i\right)}\cdot \text{Softmax(}\frac{X^{\left(i\right)}}{\mu^{\left(i\right)}}),\;\mu^{\left(i\right)}={\text{mean(X}}^{\left(i\right)}) \end{array}$$

This process is similar to the ‘query-key’ interaction operation of the self-attention mechanism, but it models the relative importance among features in a more compact and grouped manner. After the interaction calculation of all group features, they are concatenated in the channel dimension to restore the original size.

To further improve the feature stability and representational ability, PWD-EFC normalizes the concatenated feature map so that its mean is zero and its standard deviation is one, and introduces learnable parameters to enhance flexibility:


(7) $$\hat{X}=\gamma \frac{X-\mu }{\sigma +\in }+\beta$$

Among them, γ and β are trainable parameters, and ∈ is a small constant to prevent division by zero. The normalized output feature $\hat{X}$ will be jointly processed with subsequent modules to improve global feature consistency. Then, the PWD-EFC module introduces a multi-level feature reconstruction mechanism, aiming to alleviate the problem of inconsistent expression between deep semantic features and shallow detail features. In this process, the module first estimates the importance weights of different channels according to the previously generated feature maps. Specifically, the global average pooling and the Sigmoid activation function are used to generate the channel attention map:


(8) $$\begin{array}{c} {\text{w}}_{c}=\sigma (\text{AvgPool(X)} \end{array})$$

Using this weight, the fused features are divided into strong semantic features (strong features) and weak semantic features (weak features):


(9) $$\begin{array}{c} {\text{X}}_{strong}={\text{w}}_{c}\cdot \text{X},{\text{X}}_{weak}=(1-{\text{w}}_{c})\cdot \text{X} \end{array}$$

Next, to enhance the detailed expression ability of strong features, use a 1×1 convolution to perform feature transformation on them:


(10) $$\begin{array}{c} {\text{X}}_{strong}^{'}={\text{Conv}}_{1\times 1}{\text{(X}}_{strong}) \end{array}$$

At the same time, depthwise separable convolution is applied to weak features, aiming to enhance their semantic richness and reduce parameter overhead.


(11) $$\begin{array}{c} {\text{X}}_{weak}^{'}={\text{DWConv(X}}_{weak}) \end{array}$$

After improving the expressive ability of strong and weak features through the above methods respectively, the module uses element-wise addition for fusion, and finally outputs the enhanced feature map.


(12) $$X_{L}=X_{strong}^{'}+X_{weak}^{'}+\hat{X}$$

Where $\hat{X}$ is the aforementioned global semantic compensation term after standardization, which is used to improve the global consistency of the fusion result. This fusion strategy ensures the integrity of semantic expression while retaining detailed information, thus significantly enhancing the detection performance of small targets in complex backgrounds.

#### 2.7.3 D-sample down-sampling module

**Figure 11:**
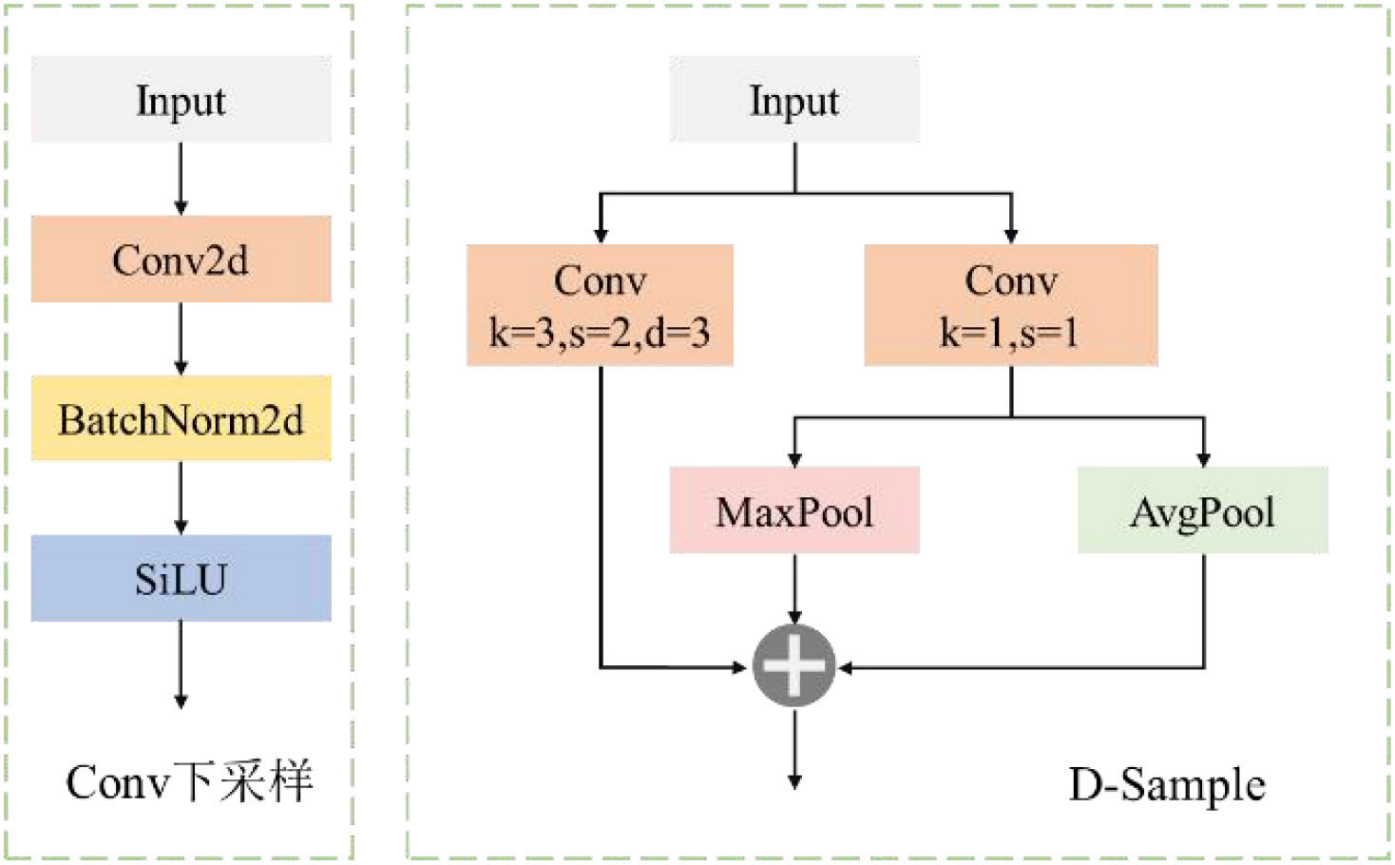
D-sample structural framework.

The main role of down-sampling is to reduce the spatial resolution of the feature map, thereby reducing the computational cost, and at the same time, extracting more robust high-level semantic information. In order to optimize the down-sampling strategy and enhance the feature representation ability, this paper designs a down-sampling module called D-Sample, whose structure is shown in **Figure 11**. Standard convolutional down-sampling achieves the down-sampling of the feature map following the order of convolution, normalization, and then activation function. However, D-Sample adopts a dual-branch structure, which further enriches the combination of feature maps and reduces the loss of tiny features.

By combining various convolutional and pooling operations, D-Sample achieves efficient information extraction and feature transformation. It aims to improve the detection accuracy of small targets of PWD while reducing computational overhead. The core idea of this module is to retain key feature information during down-sampling and enhance the model's perception ability for targets of different scales. Specifically, the module contains two main convolutional layers. Among them, the convolutional layer Conv1 uses a large kernel convolution with a stride of 2 to achieve spatial down-sampling and directly extract global features. The convolutional layer Conv2 uses a 1×1 convolutional kernel to perform channel transformation while maintaining the spatial size of the input feature map unchanged, so as to adapt to the subsequent feature fusion process. In addition, D-Sample also integrates pooling operations to further enrich the feature representation ability and improve the modeling ability for PWD targets of different scales, thereby enhancing the target detection performance of the model in complex forest environments. The input X first passes through Conv1 to obtain X1. Then, after passing through Conv2, X is divided into two parts along the channel dimension, and max-pooling and average-pooling operations are performed respectively to obtain X2 and X3. Finally, the three feature maps X1, X2, and X3 are concatenated through Concat to obtain the output of D-Sample. The operation process is as follows:


(13) $$\begin{array}{c} {\text{X}}_{1}={\text{Conv}}_{3\times 3,2}\left(\text{X};{\text{C}}_{1},\text{C}\right) \end{array}$$

(14) $$\begin{array}{c} X_{2}={\text{Maxpool}}_{3\times 3,2}\left({\text{X}}_{2}^{'};1\right) \end{array}$$

(15) $$\begin{array}{c} X_{3}={\text{Avgpool}}_{3\times 3,2}\left({\text{X}}_{3}^{'};1\right) \end{array}$$

(16) $$\begin{array}{c} Output=Concat\left({\text{X}}_{1},{\text{X}}_{2},{\text{X}}_{3}\right) \end{array}$$

Among them, Conv3×3,2 represents a convolution with a kernel size of 3×3 and a stride of 2; Maxpool_3×3,2_ and Avgpool_3×3,2_ respectively represent the maximum pooling and average pooling operations with a kernel size of 3×3 and a stride of 2; ${\text{X}}_{2}^{'}$ first-order derivative and ${\text{X}}_{3}^{'}$ first-order derivative respectively represent the two parts after the output mapping of Conv_2_.

The D-Sample down-sampling module designed in this paper can reduce the spatial size of the feature map while retaining and enhancing features of different scales and types, improving the model's ability to recognize small targets of PWD. In addition, the design of the D-Sample module takes into account both computational efficiency and detection accuracy, which helps to optimize the feature extraction ability of the lightweight LW-PWDNet model, making it more suitable for UAV edge computing devices with limited computational resources.

### 2.8 Prediction layer

This paper constructs an ultra-lightweight prediction layer, LightShiftHead, to reduce the computational complexity and improve the efficiency of PWD detection. This prediction layer introduces a parameter-free simple attention mechanism, SimAM(Yang et al., 2021), as well as DWConv and SPConv to optimize the allocation of computing resources and the model's inference speed.

**Figure 12:**
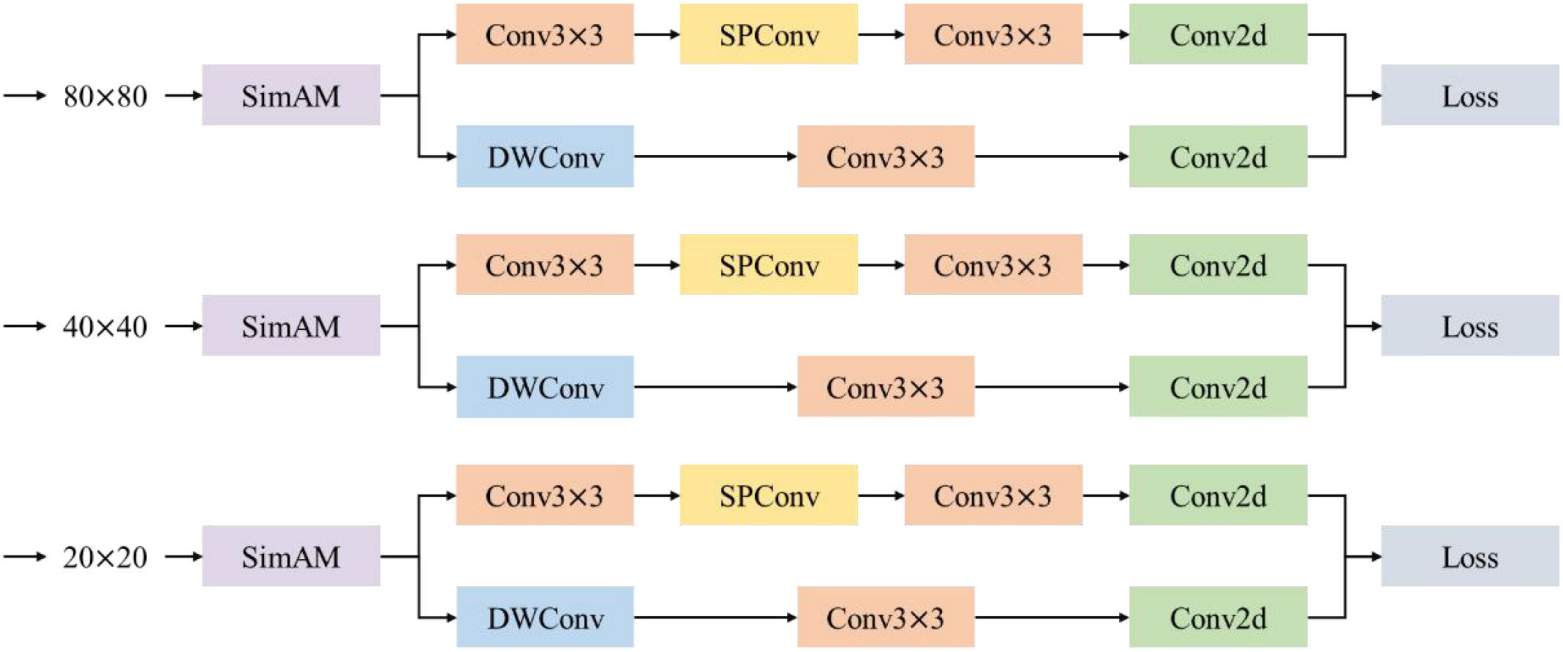
D-sample structure.

Specifically, SimAM is inserted at the front-end of the three prediction branches to enhance the feature expression ability while avoiding introducing additional computational burden. In the classification branch, DWConv is adopted to reduce the amount of computation, and in the regression branch, SPConv is introduced to further improve the efficiency of feature extraction. The structure of LightShiftHead is shown in **Figure 12**.

### 2.9 LW-PWDNet design of the model loss function

To address the diverse scenarios and high-precision requirements in PWD detection, the LW-PWDNet model adopts a composite loss function, which combines classification loss, bounding box loss, and confidence loss.

#### 2.9.1 Classification loss

General loss functions may bring certain computational burden, while cross-entropy loss is more concise in calculation and can achieve more efficient training. It is especially suitable for lightweight designs that require simplifying the calculation process and reducing the model's resource consumption. Therefore, the LW-PWDNet model in this paper adopts cross-entropy loss as the classification loss, and its calculation formula is as follows:


(17) $$\begin{array}{c} {\text{L}}_{class}=-\sum_{i-1}^{N}\sum_{c=1}^{4}{\text{y}}_{i,c}\text{log}\left(\hat{p_{i,c}}\right) \end{array}$$

Among them, N is the number of detected targets in the current batch. Suppose the category numbers c of the four infection stages of the target of PWD are 1–4 respectively. Then in the formula, ${\text{y}}_{i,c}$ is the true label of the i-th target in the c-th category, and $\hat{p_{i,c}}$ is the probability that the model predicts whether this sample belongs to category c.

#### 2.9.2 Bounding box loss

In order to improve the recognition ability of the detection model for small targets and enhance the matching accuracy between the prediction box and the ground truth box while maintaining the light weight of the model, the LW-PWDNet model adopts Scale-based Dynamic Loss (SDIoU Loss) (Su et al., 2024) in the design of the bounding box regression loss. SDIoU can dynamically adjust the impact factors of scale and position loss according to the target scale, and can better adapt to the detection of targets of different scales. Especially, it has higher sensitivity to the detection of small target PWD.

SDIoU improves the target box matching effect by comprehensively considering the Intersection over Union (IoU), aspect ratio loss, and center point offset loss. First, the loss function calculates the aspect ratio difference between the prediction box and the ground truth box, and uses the arctangent function to smooth it to form the aspect ratio loss term v. Subsequently, a proportional factor α is introduced to dynamically adjust the contribution of the aspect ratio loss at different IoU levels. In addition, SDIoU combines the area information of the target box, uses the scale factor δ and the weight term β to weight the loss of small targets to prevent small targets from being ignored during the optimization process. At the same time, in order to further improve the positioning accuracy of the target box, the loss function also adds the center point offset loss term ρ2/c2, where c is the diagonal length of the smallest enclosing rectangle of the two boxes, ensuring that the center of the prediction box has minimal deviation from the center of the ground truth box, thus enhancing detection robustness. The calculation formula of the SDIoU loss function is as follows:


(18) $$\begin{array}{c} v=\frac{4}{\pi^{2}}{\left(\tan^{-1}\frac{w_{2}}{h_{2}}-\tan^{-1}\frac{w_{1}}{h_{1}}\right)}^{2} \end{array}$$

(19) $$\begin{array}{c} \alpha =\frac{v}{v\;-\;IoU\;+\;\left(1\;+\;є\right)} \end{array}$$

(20) $$\begin{array}{c} \beta =\frac{w_{2}h_{2}\delta }{81} \end{array}$$

(21) $$\begin{array}{c} \beta =\{\begin{array}{c} \delta ,\;\;if\;\beta >\delta \\ \beta ,\;\;otherwise \end{array} \end{array}$$

(22) $$\begin{array}{c} L_{SDIoU}=\delta -\beta +\left(1-\delta +\beta \right)\left(IoU-v\alpha \right)-\left(1+\delta -\beta \right)\frac{\rho^{2}}{c^{2}} \end{array}$$

Among them, w_1_, h_1_ and w_2_, h_2_ represent the widths and heights of the ground truth box and the predicted box respectively. ∈ is a very small positive number to prevent the denominator from being zero. ρ^2^ represents the square of the Euclidean distance between the center points of the predicted box and the ground truth box, and c^2^ represents the square of the diagonal length of the minimum bounding rectangle of the predicted box and the ground truth box.

In summary, the calculation formula of the loss function of the LW-PWDNet model is as follows:


(23) $$\begin{array}{c} L_{LW}=\lambda_{1}L_{class}+\lambda_{2}L_{BCE}+\lambda_{3}L_{SDIoU} \end{array}$$

For the proposed composite loss function, the weights λ_1_, λ_2_, and λ_3_ are empirically assigned according to the relative importance of classification, confidence, and bounding box regression. In this study, λ_1_ = 0.5, λ_2_ = 0.3, and λ_3_ = 0.2, which were determined based on preliminary experiments to balance detection accuracy and convergence stability.

## 3 Experimental results

### 3.1 Experimental details

The hardware and software configurations, model training parameters, and experimental environment are as follows. The hardware setup includes an NVIDIA RTX 4070 GPU and a 13th Gen Intel(R) Core(TM) i7-13700KF processor. The software environment is based on Python 3.9, PyTorch 2.0.1, and CUDA 12.6 for GPU acceleration. For model training, the initial learning rate was set to 0.001 with cosine annealing adjustment, the optimizer was SGD, and the batch size was 16. All models were trained and validated on the same PWD dataset to ensure the reliability and comparability of results.

### 3.2 Comparison of experimental results and analysis

In order to comprehensively evaluate the performance of the lightweight model LW-PWDNet proposed in this paper in the detection task of PWD, this paper selects some mainstream lightweight object detection networks for comparative experiments, including the SSD model using MobileNetv2 as the backbone network, EfficientDet with an efficient feature fusion mechanism, as well as the lightweight versions YOLOv5n and YOLOv10n in the YOLO series. The results of the comparative experiments are shown in **Table 3**.

**Table 3:** Performance comparison among different lightweight models.

Model	Backbone	Stage	Evaluation indicators					
			P↑	R↑	mAP50↑	FPS↑	GFLOPs↓	Parameters↓
			(%)	(%)	(%)		(G)	(M)
SSD	MobileNetv2	PWD-E	60.5	56.2	63.2	**186.7**	**1.1**	5.4
		PWD-M	70.2	65.4	72.1			
		PWD-L	70.3	72.8	74.3			
		PWD-D	72.4	69.7	72.8			
		ALL	68.4	66.0	70.6			
EfficientDet	EfficientNet-D1	PWD-E	68.9	59.3	75.6	49.3	6.3	6.6
		PWD-M	78.6	75.4	82.6			
		PWD-L	79.2	78.6	88.1			
		PWD-D	77.3	79.3	82.4			
		ALL	76.0	73.1	82.2			
YOLOv5n	CSPResNet	PWD-E	71.2	78.9	80.1	183.4	4.7	**1.9**
		PWD-M	80.1	77.6	88.6			
		PWD-L	85.3	89.8	90.3			
		PWD-D	76.3	88.5	88.4			
		ALL	78.2	83.7	86.9			
YOLOv10n	EfficientFormerV2	PWD-E	**73.4**	81.4	81.2	169.2	8.1	3.1
		PWD-M	81.8	75.2	80.6			
		PWD-L	86.1	87.6	92.1			
		PWD-D	77.6	87.9	88.2			
		ALL	79.7	83.0	85.5			
LW-PWDNet	GH-Backbone	PWD-E	73.3	**81.7**	**83.4**	166.8	5.6	**1.9**
		PWD-M	**88.4**	**88.7**	**91.8**			
		PWD-L	**87.6**	**89.4**	**93.6**			
		PWD-D	**78.6**	**90.1**	**90.1**			
		ALL	**82.0**	**87.5**	**89.7**			

Bold values indicate the best results for each metric among the compared models.

Based on the data in **Table 3**, the following observations can be made:

1. In terms of detection accuracy, compared with other lightweight models, LW-PWDNet demonstrates superior sensitivity to PWD lesions. Its mAP index reaches 89.7%, and the detection accuracy for the early-stage infection reaches 83.4%. The detection accuracies for the mid-stage, late-stage, and dead-tree infection stages all exceed 90%. Among the YOLO series of models, YOLOv10n has a better perception ability for early-stage PWD than YOLOv5n, but its overall mAP is 1.4% lower than that of YOLOv5n. This may be attributed to YOLOv10n's focus on speed optimization, which involves more aggressive pruning of network parameters or simplification of feature extraction modules. These trade-offs slightly compromise the model's ability to capture complex contextual information, thereby leading to a marginally inferior global detection performance compared with YOLOv5n. The EfficientDet model generally has a good detection accuracy, but its detection accuracy for early-stage small-target PWD is still low, with an mAP of only 75.6%. SSD performs poorly in the detection tasks of each PWD infection stage, especially for the early-stage infection, with an mAP of only 63.2%, indicating limited sensitivity to PWD symptoms.
2. In terms of recall rate, the overall recall rate of LW-PWDNet reaches 87.5%, which is 3.8% and 4.5% higher than the relatively well-performing YOLOv5n (83.7%) and YOLOv10n (83.0%) models respectively. This indicates that LW-PWDNet has a lower missed-detection rate in the PWD detection task and performs more stably in the PWD target detection task. Especially in the early-stage infection, its recall rate reaches 81.7%, which has more advantages than other models and helps to improve the effectiveness of early-stage PWD prevention and control.
3. In terms of model computational complexity and inference speed, the GFLOPs of the LW-PWDNet model is 5.6G, which is much lower than that of YOLOv10n (8.1G), slightly higher than that of YOLOv5n (4.7G), and comparable to that of EfficientDet (6.3G). However, LW-PWDNet performs relatively well in inference speed, with an FPS of 166.8, only slightly lower than that of YOLOv10n (169.2). The EfficientDet model is limited by its large computational complexity (GFLOPs of 6.3G) and deep network structure, with an FPS of only 49.3, making it difficult to meet the requirements of real-time detection.
4. In terms of model parameter quantity, the parameter quantity of the LW-PWDNet model is only 1.9M, the same as that of YOLOv5n. In contrast, the parameter quantities of YOLOv10n, EfficientDet, and SSD are all higher than 1.9M. Especially, due to the adoption of a more complex backbone network, the parameter quantity of EfficientDet increases significantly, resulting in a decrease in inference speed. While maintaining high detection performance, LW-PWDNet effectively controls the parameter scale of the model, further improving its applicability in resource-constrained environments.

Comprehensive analysis shows that LW-PWDNet achieves high detection accuracy and recall rate in different infection stages, especially in the mid-stage and late-stage infection stages, its detection performance is significantly better than that of the comparison models. At the same time, LW-PWDNet maintains a computational complexity comparable to that of YOLOv10n and performs outstandingly in parameter quantity control, with only 1.9M, making it suitable for edge-computing devices. Although its inference speed is slightly lower than that of YOLOv5n, it has obvious advantages in detection performance.

**Figure 13:**
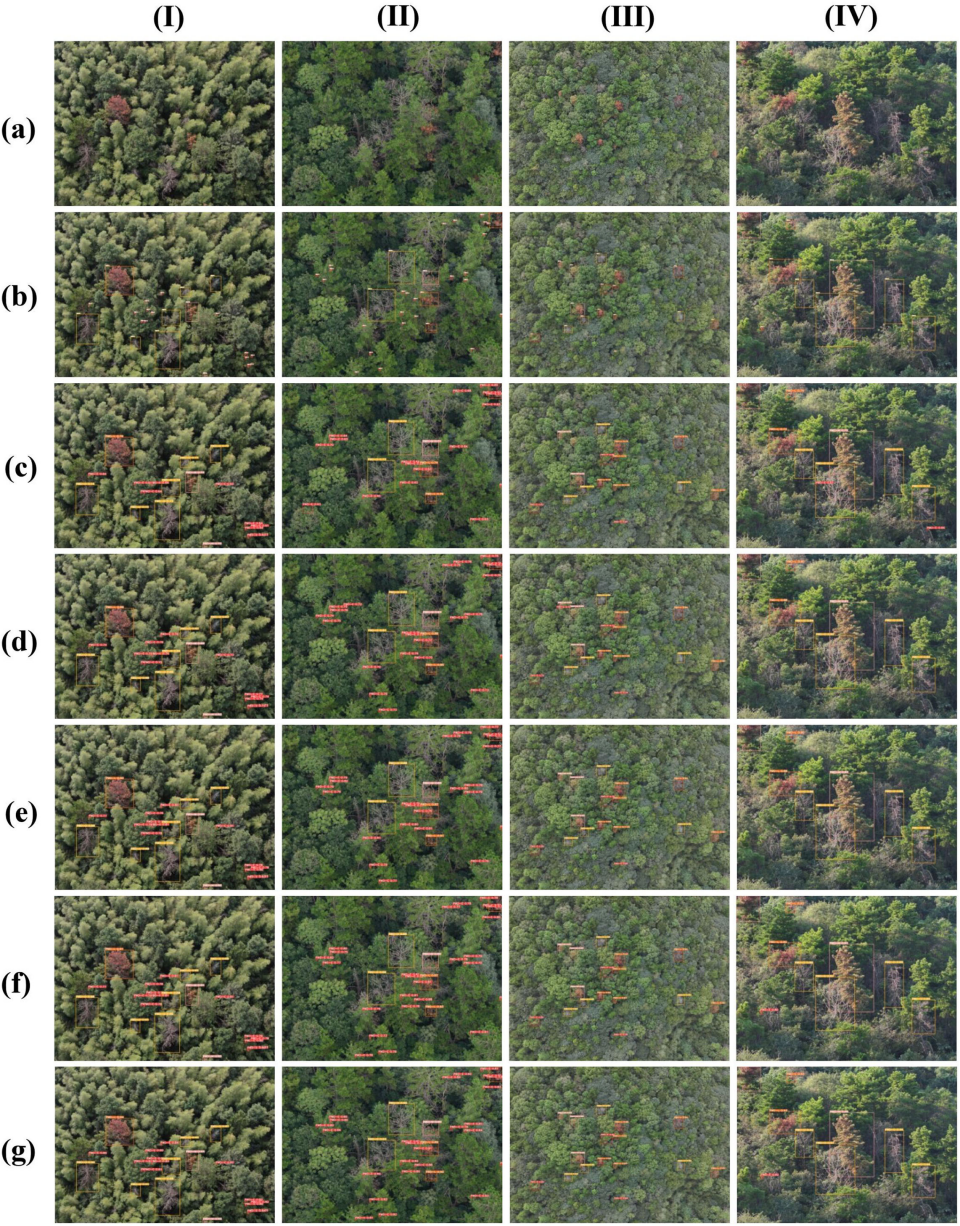
Detection effects of different models on the small target PWD dataset. **(a)** Original image, **(b)** Ground Truth, **(c)** SSD, **(d)** EfficientDet, **(e)** YOLOv5n, **(f)** YOLOv10n, **(g)** LW-PWDNet.

In order to visually compare the performance of each model in the PWD detection task, this paper selects 4 representative images from the constructed PWD dataset and conducts a visual analysis of the detection results of each model. The detection effects of each model are shown in the **Figure 13**. These 4 images cover different infection stages (early, mid, late, dead tree), complex background environments (dense forest areas, sparse forest areas, mountainous environments), and different shooting angles (vertical overhead shooting and oblique view) to comprehensively evaluate the detection effects of each model. Among them, **Figure 13** (I) and (II) are vertical overhead shooting images obtained by the drone at the same flight altitude in different forest areas. **Figure 13** (III) and (II) are vertical overhead shooting images at different flight altitudes in the same forest area, and **Figure 13** (IV) and (II) are oblique-view images at the same height and different angles.

From the detection results in **Figure 13**, it can be seen that LW-PWDNet and YOLOv10n perform excellently in the PWD target detection task. They can not only accurately detect all infected targets, but also have a strong ability to distinguish different infection stages, demonstrating high detection accuracy and stability. In contrast, YOLOv5n has a certain degree of missed detection when detecting early PWD small targets. A total of 3 missed detections occurred in the test samples, and its sensitivity to the early infection stage is relatively low. SSD and EfficientDet perform relatively poorly in the PWD detection task, with relatively prominent problems of missed detection and false detection. Among them, SSD had a total of 16 missed detections and 5 false detections. It is particularly vulnerable to interference in complex background environments, resulting in some withered broad-leaved trees being misidentified as infected areas. Although the overall detection accuracy of EfficientDet is better than that of SSD, it still has 5 missed detections and 3 false detections, and there are still deficiencies in its detection ability for small PWD targets. Overall, LW-PWDNet shows high detection reliability and robustness in complex forest environments and different stages of PWD infection.

### 3.3 Results and analysis of ablation experiments

To verify the impact of each key module of LW-PWDNet on the detection performance of small-target PWD, this paper designs multiple groups of ablation experiments, and analyzes them from four aspects: mAP, GFLOPs, Parameters, and FPS. The experiments successively evaluate the contributions of RE-Block, PWD-EFC and D-Sample in the feature fusion layer, and LightShiftHead in the prediction layer to the model performance. Among them, Model 1 serves as the baseline model, using only the GH-Backbone backbone network without including all the above modules. The results of the ablation experiments are shown in **Table 4**.

**Table 4:** Results of the ablation experiments of the LW-PWDNet model.

Model	RE-block	PWD-EFC	D-sample	LightShiftHead	Stage	Evaluation indicators			
						mAP50↑	FPS↑	GFLOPs↓	Parameters↓
						(%)		(G)	(M)
1					PWD-E	78.4	173	6.4	2.3
					PWD-M	83.6			
					PWD-L	88.7			
					PWD-D	86.3			
					ALL	84.3			
2	✓				PWD-E	80.2	160	5.7	2.1
					PWD-M	87.3			
					PWD-L	91.6			
					PWD-D	87.6			
					ALL	86.7			
3	✓	✓			PWD-E	81.4	175	5.7	2.2
					PWD-M	89.6			
					PWD-L	91.8			
					PWD-D	87.9			
					ALL	87.7			
4	✓	✓	✓		PWD-E	82.5	172	5.6	2.0
					PWD-M	90.7			
					PWD-L	92.8			
					PWD-D	88.3			
					ALL	88.6			
5	✓	✓	✓	✓	PWD-E	83.4	166	5.6	1.9
					PWD-M	91.8			
					PWD-L	93.6			
					PWD-D	90.1			
					ALL	89.7			

According to the quantitative results of the ablation experiments in **Table 4**, the following conclusions can be drawn:

1. RE-Block can effectively enhance the feature extraction ability while reducing the computational cost, and improve the detection ability of the lightweight model for small-target PWD. After introducing RE-Block, the GFLOPs of the model decreased from 6.4G to 5.7G, and the number of parameters decreased by 0.2M, indicating that both the computational complexity and storage requirements were optimized. At the same time, the detection accuracy of the model at each infection stage was significantly improved, especially in the early infection stage (mAP increased from 78.4% to 80.2%), and the overall mAP increased by 2.4%.
2. The PWD-EFC designed in this paper improves the correlation between features at different levels by enhancing spatial attention and channel context information, and further enhances the model's detection ability for PWD targets of different scales. After adding PWD-EFC, the model inference speed was significantly improved (FPS increased from 160 to 175), while the GFLOPs and the number of parameters remained basically unchanged. However, the detection accuracy was further improved, especially in the early (+1.2%) and middle (+2.3%) infection stages, and the overall mAP increased to 87.7%. Although the introduction of the PWD-EFC module results in a negligible parameter increase (only 0.1M), the computational complexity (GFLOPs) remains unchanged, while the detection accuracy improves by 1.0%. This indicates that PWD-EFC maintains a lightweight design while achieving significant performance gains.
3. The innovatively designed D-Sample in this paper can more effectively aggregate multi-scale information during the feature fusion process, improve the detection ability for PWD targets, especially for early-infected targets, and further compress the model scale, making it more suitable for the edge-computing environment. After adding the D-Sample down-sampling module proposed in this paper, the number of parameters of the model decreased from 2.2M to 2.0M, and the GFLOPs decreased to 5.6G, indicating that D-Sample optimized the model structure while reducing the computational cost. At the same time, the detection accuracy at each stage was improved to varying degrees, with the mAP in the early infection stage increasing to 82.5% and the overall mAP reaching 88.6%.
4. After introducing the lightweight self-attention mechanism LightShiftHead, the GFLOPs and the number of parameters of the model remained unchanged, but the overall detection accuracy was further improved (mAP increased from 88.6% to 89.7%). Especially, the detection accuracy in the early infection stage increased to 83.4%, and the mAP in the middle, late, and dead-tree infection stages increased by 1.1%, 0.8%, and 1.8% respectively. Although LightShiftHead slightly decreased the inference speed (FPS decreased from 172 to 166), it can effectively enhance the feature representation ability, improve the model's discrimination ability for PWD targets in complex forest environments, and further reduce the missed-detection rate.

**Figure 14:**
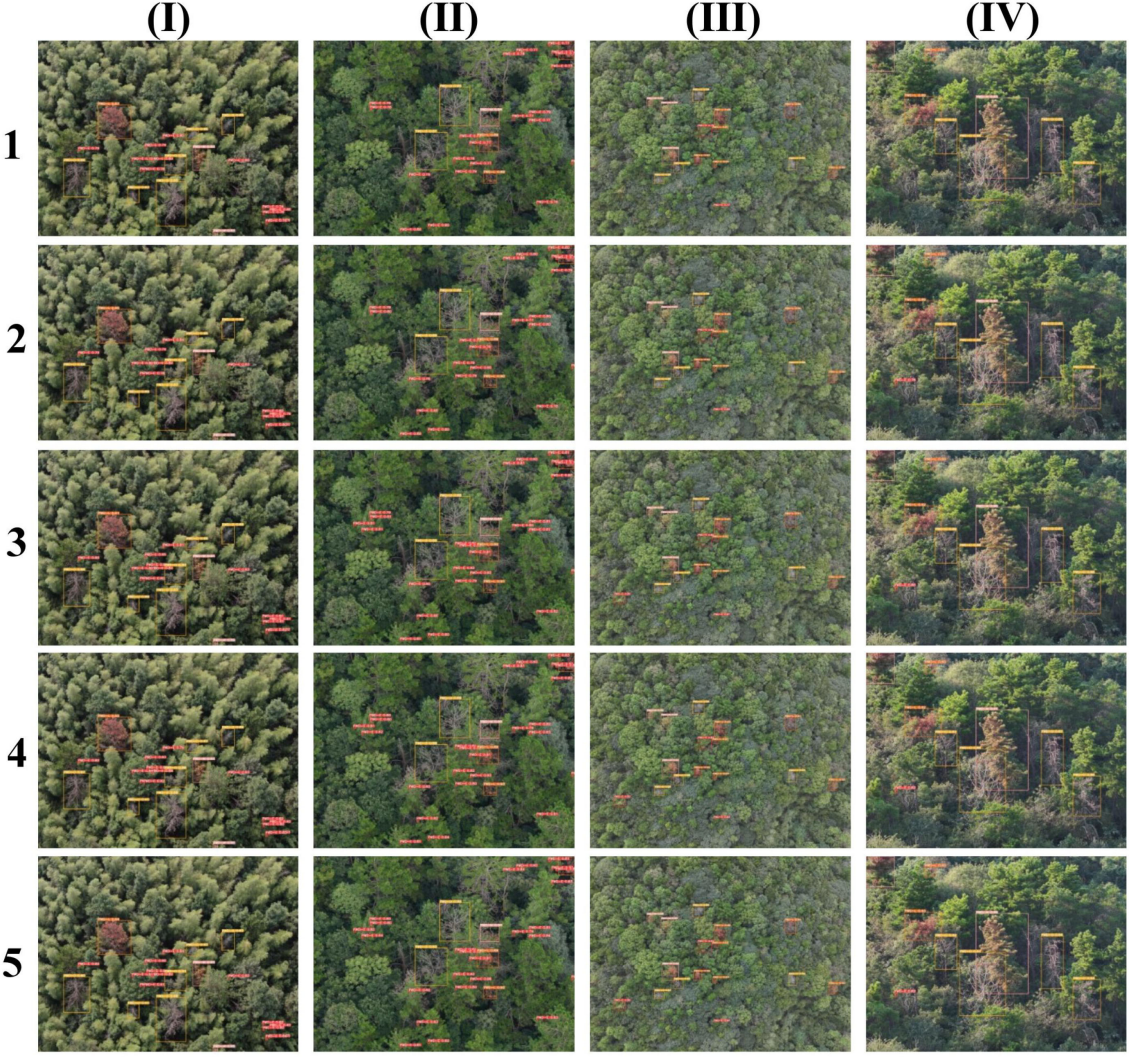
Comparison chart of ablation experiments. (1) GH-Backbone. (2) GH-Backbone +RE-Block. (3) GH-Backbone+RE-Block+PWD-EFC. (4) GH-Backbone+RE-Block+PWD-EFC+D-Sample. (5) GH-Backbone+RE-Block+PWD-EFC+D-Sample+LightShiftHead.

The results of the ablation experiment are shown in the **Figure 14**.

According to the visual analysis of the detection results in the **Figure 14**, in the test set, there are a total of 9 undetected samples in Model 1, and all of them are PWD individuals in the early stage. This phenomenon is mainly attributed to the small target size and unclear texture features of early PWD trees in the images, which increases the detection difficulty. In contrast, the number of undetected samples in Model 2 is reduced to 8, and the detection accuracy is significantly improved compared with Model 1. The number of undetected samples in Model 3 and Model 4 further decreases to only 2, and there is also a certain increase in detection accuracy. Finally, Model 5 is superior to the previous models in terms of both the undetected rate and detection accuracy, showing a significant performance improvement compared with Model 1. This result indicates that LW-PWDNet has higher accuracy and stronger stability in the early PWD detection task, especially in dealing with small-target detection scenarios.

In conclusion, the LW-PWDNet model designed in this paper, through a variety of lightweight structures and strategies to enhance feature extraction, improves the detection ability for small-target PWD while ensuring real-time detection. It is suitable for resource-constrained edge-computing environments and can provide an efficient and reliable solution for the early monitoring of PWD.

### 3.4 Cross-regional generalization analysis

To evaluate the robustness and transferability of the proposed lightweight model LW-PWDNet in practical deployment scenarios, a cross-regional validation experiment was designed. Specifically, UAV-acquired images from two regions with distinct geographical and ecological characteristics were utilized: Liyang City (Jiangsu Province) and Qianshan City (Anhui Province). These regions differ in forest composition, canopy density, terrain morphology, and lighting conditions. The two regions exhibit distinct ecological and environmental characteristics, introducing natural variability that challenges model generalization. Region A is characterized by a diverse vegetation composition, including coniferous forests, evergreen broadleaf forests, deciduous broadleaf forests, and artificial mixed plantations. The forest structure is relatively complex, with heterogeneous canopy layers and moderate variability in canopy density. The terrain is dominated by gently undulating hills, while lighting conditions are generally stable due to relatively open topography and fewer terrain-induced shadows. In contrast, Region B is characterized by mixed natural forests with high species diversity, where PWD-susceptible pines are interspersed among other tree species. The canopy is highly heterogeneous and denser in patches, and the mountainous terrain leads to complex shading effects. Moreover, lighting conditions in Region B are less stable, with frequent shadow occlusion and strong variability in illumination angles caused by rugged terrain and slope orientation. These differences in forest composition, canopy density, terrain morphology, and lighting introduce domain shifts that may significantly affect the robustness of PWD detection models.

The dataset from Region A contained 11,126 UAV images with 8,500 annotated pine trees. Among them, early-stage PWD trees (PWD-E) accounted for 44.7%, followed by middle-stage (PWD-M, 27.1%), late-stage (PWD-L, 16.5%), and dead trees (PWD-D, 11.7%). The dataset from Region B included 8,420 UAV images with 7,200 annotated pine trees, of which early-stage PWD trees made up 44.4%, while PWD-M, PWD-L, and PWD-D accounted for 27.8%, 16.7%, and 11.1%, respectively. The relatively high proportion of early-stage infections in both datasets ensured a robust evaluation of the proposed model’s ability to detect small targets under cross-regional conditions.

In this experiment, models were trained using data from Region A and tested on data from Region B, thereby simulating deployment under domain shift conditions. For comparative analysis, the same five lightweight detection models as employed in the comparative experiments were selected as controls, including SSD with MobileNetV2 backbone, EfficientDet with EfficientNet-D1 backbone, YOLOv5n with CSPResNet backbone, YOLOv10n with EfficientFormerV2 backbone, and the proposed LW-PWDNet. The evaluation metric adopted was mAP, and statistical analyses were performed on the detection performance of each model on both the source domain test set and the target domain test set, respectively.

**Figure 15:**
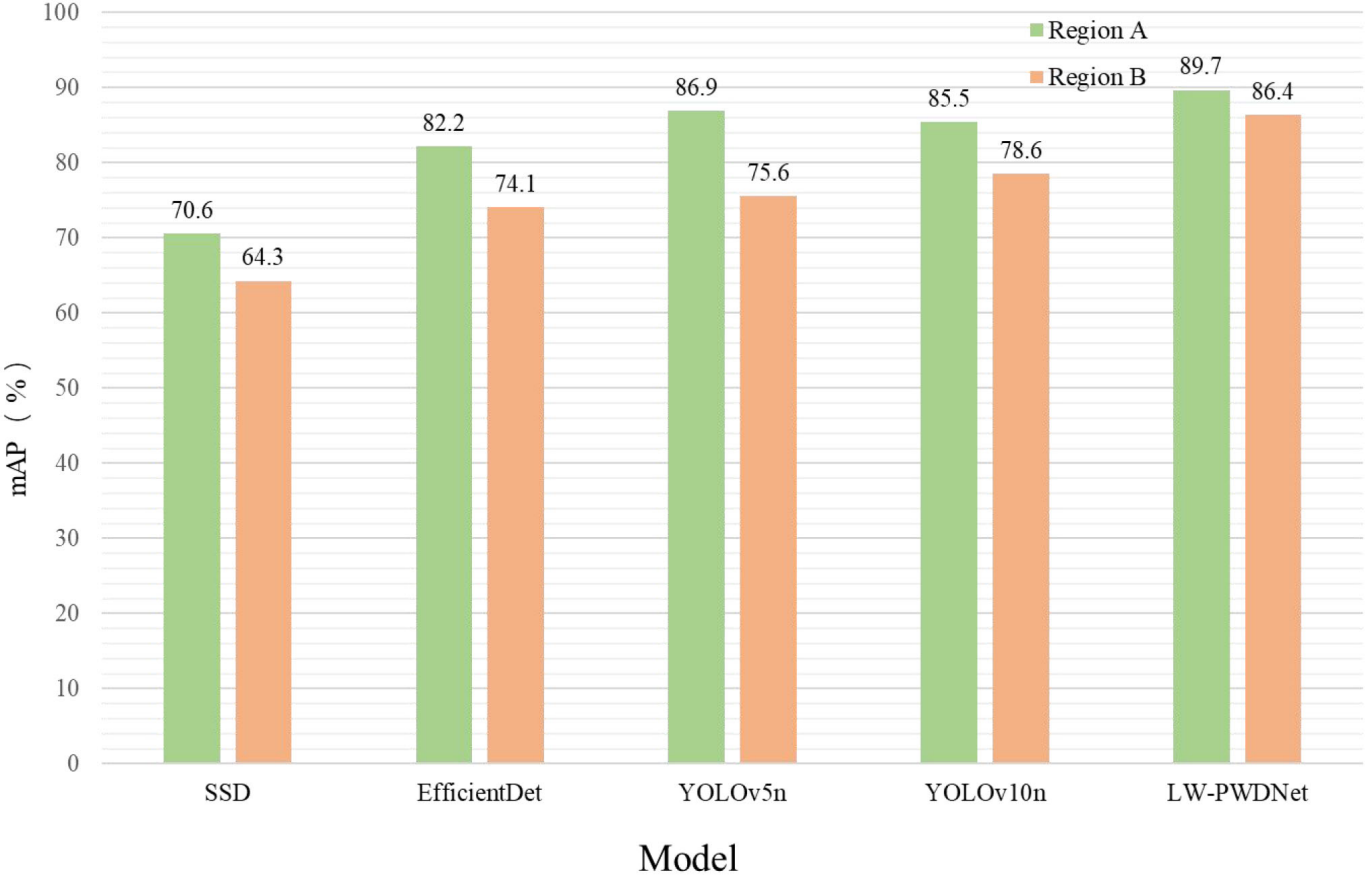
Cross-region verification mAP comparison chart.

/The results, as shown in the **Figure 15**, indicate that although all models experienced a certain degree of performance decline when migrating from the source region to the target region, there are significant differences in the extent of the decline. The mAP of the SSD model decreased from 70.6% to 64.7%, a drop of 5.9 percentage points; EfficientDet decreased from 82.4% to 75.2%, a drop of 7.2 percentage points; YOLOv5n decreased from 88.9% to 76.4%, a drop of 12.5 percentage points; YOLOv10n decreased from 86.6% to 80.1%, a drop of 6.5 percentage points. In contrast, the LW-PWDNet model decreased from 91.8% to 88.6%, with a decline of only 3.2%. It is not only the model with the smallest decline among the five models but also the model with the highest absolute accuracy in the target region.

This result fully demonstrates that LW-PWDNet exhibits remarkable stability and generalization ability when facing cross-regional forest environmental differences. A smaller decline in accuracy means that this model has a stronger adaptability to changes in forest stand structure, lighting conditions, and background interference, which is of great significance for building a cross-regional and long-term operating PWD monitoring system.

## 4 Discussion

The PWD is characterized by rapid spread and high lethality. It only takes dozens of days for an infected tree to die. Therefore, early, accurate, and low-cost monitoring methods are crucial for curbing the spread of the epidemic. Traditional ground survey methods are inefficient and difficult to meet the needs of large-scale real-time monitoring. Satellite remote sensing is very difficult to monitor small targets of PWD. In recent years, with the development of UAV remote sensing and object detection algorithms, researchers have gradually applied deep learning technology to the automatic identification of diseased trees. In particular, lightweight small object detection methods have become a key direction for achieving accurate monitoring of PWD. For the above reasons, the lightweight small object PWD detection model LW-PWDNet proposed in this paper demonstrates excellent recognition accuracy in the early monitoring of PWD.

This paper proposes a lightweight small-target PWD detection model, LW-PWDNet, aiming to reduce the model's computational complexity while enhancing the detection ability of small-target PWD diseases. First, in this chapter, a lightweight backbone network, GH-Backbone, is designed in the backbone network layer, and the HGNetV2 network structure is reconstructed. This not only reduces the computational complexity but also improves the perception ability of the PWD disease area. Second, in the feature fusion layer, this chapter designs the RE-Block. Under the premise of ensuring the effective fusion of PWD target information at different scales, the computational complexity is reduced. At the same time, a lightweight feature fusion module, PWD-EFC, is designed in the feature fusion layer to improve the correlation between features at different levels. Moreover, a lightweight down-sampling module, D-Sample, is designed to effectively enhance the model's feature expression ability for multi-scale PWD targets. In addition, this chapter constructs an ultra-lightweight prediction layer, LightShiftHead, to further improve the detection accuracy of small-target PWD. The effectiveness of LW-PWDNet is evaluated through ablation experiments and comparative experiments. The experimental results show that compared with the benchmark model, the method in this chapter significantly improves the detection accuracy of PWD targets while maintaining a high inference speed, especially in the detection ability of small targets in the early infection stage. In addition, visual analysis further validates the robustness of LW-PWDNet in the complex forest area background, demonstrating its potential in practical applications.

**Figure 16:**
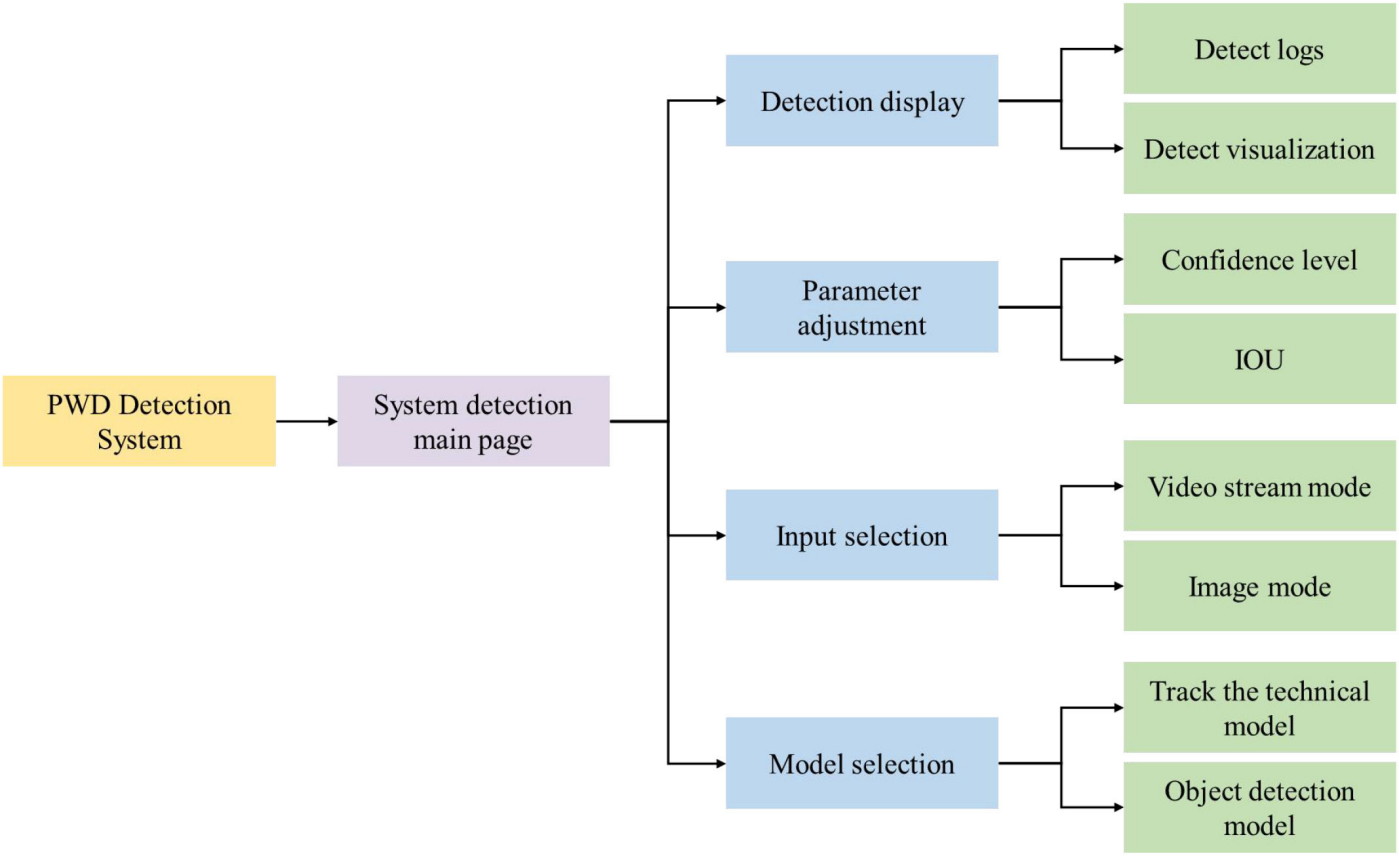
Architecture of pine wilt disease detection system.

To further validate the practicality of the proposed LW-PWDNet, we implemented a lightweight graphical detection system based on PyQt5, integrating both static image detection and real-time video stream tracking modules. The system provides a user-friendly interface, supports adaptive parameter adjustment, and enables real-time visualization of detection and tracking results. The overall architecture of the system is shown in **Figure 16**.

**Figure 17:**
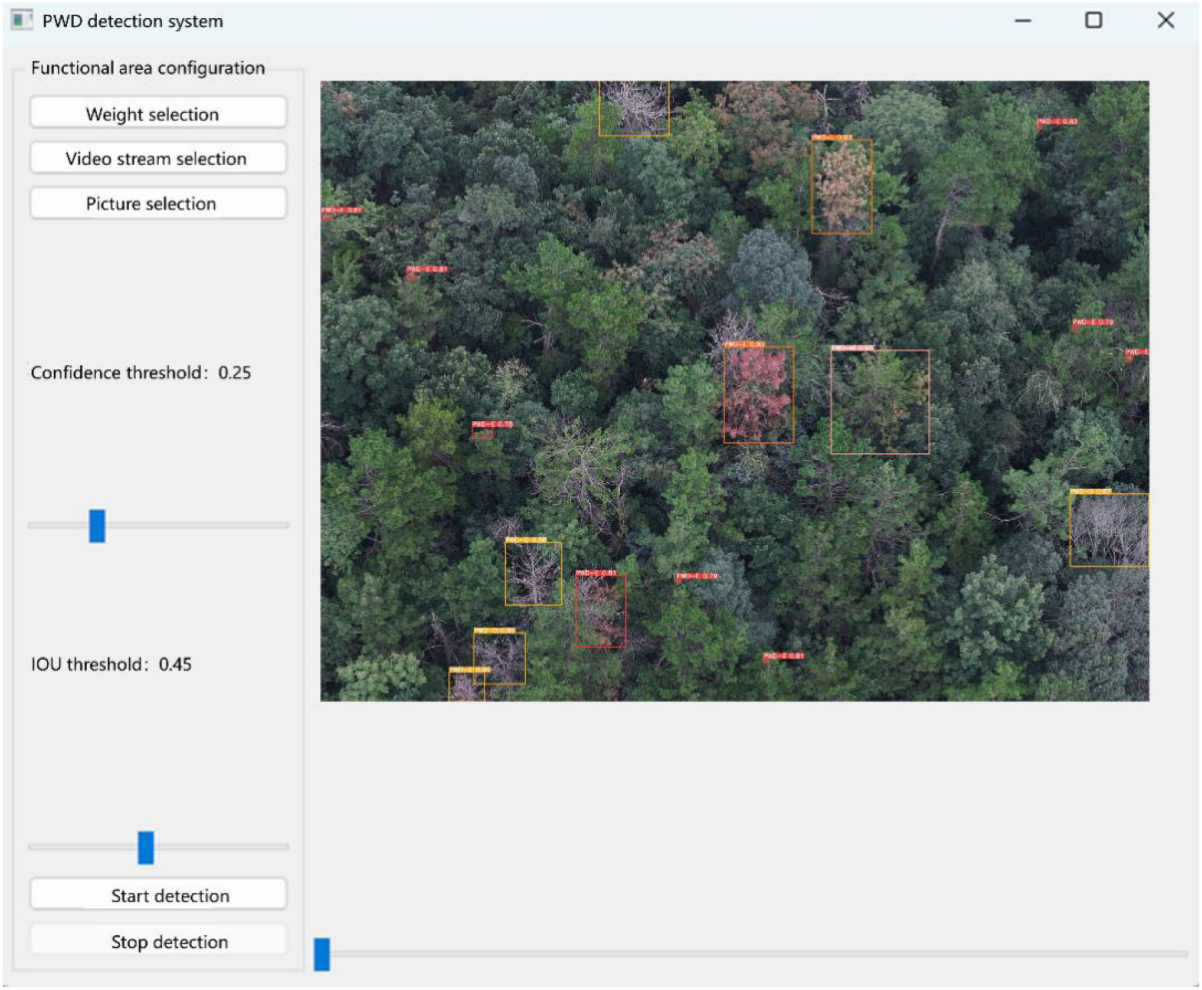
Schematic diagram of tracking count test results.

The system interface is mainly composed of two parts: the function setting area and the detection display area. In the function setting area, users can perform model selection, input method setting, and parameter adjustment. The system provides a model selection function, supporting the loading of different detection models, including object detection models and tracking and counting models, to meet the detection requirements of PWD-infected wood in different scenarios. Users can click the input selection button to choose to upload pictures for detection, or click the video detection button for object tracking and counting. In addition, the system provides a parameter adjustment function, including the adjustment of the IoU threshold and confidence threshold. Users can adjust according to the actual detection environment to optimize the detection accuracy and object tracking effect. In the detection display area, the system will present the detection results in real-time, including the detected target categories of PWD, location information, and confidence scores, and display the object detection boxes and tracking paths in a visual way. For the tracking and counting of PWD in the death stage, the system uses an object tracking algorithm to ensure accurate identification of PWD in the death stage and calculate the number of targets in the death stage to assist the forestry department in scientific evaluation and the formulation of prevention and control measures. After users open the main interface of the PWD system, they select the weight files in the function area configuration, including object detection weights and tracking and counting weights. When clicking the video stream selection button, select the video stream to be detected, and click the start detection button to start the video stream tracking and counting. The visual tracking and counting results will be displayed on the right side of the interface. The detection results are shown in **Figure 17**.

LW-PWDNet has been successfully deployed on edge devices onboard UAV platforms, where real-time inference and visualization were achieved without compromising detection accuracy. This demonstrates that the proposed model is not only theoretically effective but also practically deployable in UAV-based forest health monitoring scenarios.

Despite the strong performance of the proposed LW-PWDNet model across various stages of PWD infection, several challenges remain when deployed in real-world forest environments. In particular, occlusions, shadow interference, and uneven illumination caused by complex forest structures significantly affect the accurate identification of early-stage infected trees. In dense forests or conifer-dominated canopies, infected branches are often partially or fully blocked by upper foliage layers or affected by limited imaging angles, leading to reduced detection accuracy and a higher rate of missed detections. Furthermore, due to the subtle differences in color and texture between early-infected and healthy trees in the spatial domain, RGB imagery alone is highly susceptible to environmental variations, limiting its effectiveness in capturing the weak visual signals of early infections.

To address these limitations, multimodal sensing and deep feature fusion strategies have emerged as promising solutions for enhancing model robustness. On one hand, multispectral imagery (e.g., red-edge, near-infrared, and shortwave infrared bands) offers rich spectral cues that reflect subtle physiological and biochemical changes in tree tissues, enabling more discriminative identification of early infections. On the other hand, thermal infrared imagery and LiDAR data can provide complementary information, such as heat stress response and structural geometry, which is especially beneficial for detecting occluded or partially visible infected trees and reconstructing detailed canopy structures.

With the advancement of cross-modal deep learning models, such as those leveraging attention mechanisms and multi-scale Transformer architectures, multimodal neural networks are increasingly capable of learning synergistic representations and aligning semantics across heterogeneous data sources. This enables more effective detection in complex forest environments. To integrate these multimodal data into the existing LW-PWDNet framework, future work will focus on developing cross-modal attention mechanisms: specifically, RGB, multispectral, and LiDAR data will be fed into modality-specific lightweight encoders (extended from the GH-Backbone) to extract modality-aware features, which will then be fused via a cross-modal attention module. This module will dynamically weight the contributions of each modality based on contextual relevance (e.g., emphasizing LiDAR-derived structural features in occluded regions or multispectral signals for early-stage spectral anomalies), while maintaining the model's lightweight design to suit edge computing scenarios.

In summary, the integration of multimodal data acquisition and cross-domain feature fusion mechanisms is essential to improving the accuracy and generalization capability of early PWD detection under real-world occlusions and illumination variability. Future work should focus on developing UAV-based multisource data collection frameworks, efficient modality alignment algorithms, and lightweight multimodal fusion networks, thereby enabling stable, real-time, and high-precision detection of infected trees in complex forested areas.

## 5 Conclusion

In order to meet the real-time detection requirements in the forest resource-constrained environment and improve the detection ability of small target PWD disease areas, this paper proposes a lightweight small target PWD detection model, LW-PWDNet. The experimental results show that LW-PWDNet achieves the highest detection accuracy (mAP 89.7%) while maintaining a low computational complexity (GFLOPs 5.6G, parameter quantity 1.9M) and a high inference speed (FPS 166). Specifically, the detection accuracy in the early infection stage reaches 83.4%, in the middle infection stage reaches 91.8%, in the late infection stage reaches 93.6%, and in the dead tree stage reaches 90.1%. It can provide an efficient and lightweight detection solution for PWD in resource-constrained scenarios such as UAV inspections.

## Data Availability

The raw data supporting the conclusions of this article will be made available by the authors, without undue reservation.
